# Synthesis of thiazole-integrated pyrrolotriazinones: evaluations of cytotoxicity and effects on PI3K levels in cancer cells

**DOI:** 10.55730/1300-0527.3723

**Published:** 2025-01-20

**Authors:** Eylem KUZU, Ege ARZUK, Fuat KARAKUŞ, Burak KUZU, Hasan GENÇ

**Affiliations:** 1 Chemistry Section, Institute of Natural and Applied Sciences, Van Yüzüncü Yıl University, Van, Turkiye; 2 Department of Pharmaceutical Toxicology, Faculty of Pharmacy, Ege University, İzmir, Turkiye; 3 Department of Pharmaceutical Toxicology, Faculty of Pharmacy, Van Yüzüncü Yıl University, Van, Turkiye; 4 Department of Pharmaceutical Chemistry, Faculty of Pharmacy, Van Yüzüncü Yıl University, Van, Turkiye; 5 Department of Science, Faculty of Educational Sciences, Van Yüzüncü Yıl University, Van, Turkiye

**Keywords:** Anticancer effect, molecular docking, PI3K, pre-ADMET, pyrrolotriazinone

## Abstract

The synthesis of novel heterocyclic compounds, particularly those targeting critical signaling pathways in cancer, represents a promising approach to drug development. In this study, we designed and synthesized a series of thiazole-integrated pyrrolotriazinone derivatives, aiming to combine the antiproliferative properties of thiazole with the PI3K inhibitory activity of pyrrolotriazinones. The PI3K pathway, which plays a critical role in regulating cell growth, proliferation, and survival, is frequently dysregulated in cancer, making it an attractive target for therapeutic intervention. The synthesized derivatives were evaluated for their cytotoxic activities against MCF-7, A549, and HepG2 cancer cell lines. Their effect on PI3K protein levels was assessed to evaluate their potential as PI3K inhibitors. Preliminary results indicate that these thiazole-pyrrolotriazinone hybrids exhibit significant cytotoxic effects and may reduce PI3K protein levels in cancer cells. Furthermore, drug-likeness assessments and pre-ADMET evaluations demonstrated that the compounds exhibited promising characteristics, supporting their potential as viable drug candidates. Overall, this study highlights the potential of these novel compounds in cancer therapy and provides valuable insights into the design of small molecules that can target key regulatory pathways involved in cancer progression.

## 1. Introduction

Methods for the development of heterocyclic compounds have reached a significant level of sophistication; however, the therapeutic efficacy and various pharmacological properties of these compounds remain insufficient for effectively targeting the biological processes underlying many unresolved diseases [1]. A deeper understanding of these biological processes and the identification of new compounds capable of modulating them continues to be a vital area of research, particularly in the discovery of novel heterocyclic structures [2]. In the context of modern drug design, small molecules, especially heterocyclic compounds, continue to provide promising starting points for drug development [3]. In contemporary pharmaceutical research, given the emergence of new diseases, drug resistance, and unresolved pathogenic processes, compounds with unconventional structural features are increasingly favored in drug design. Moreover, in the current era of drug development, selecting target diseases and developing drug candidates that specifically target pathogenic signaling pathways have become fundamental priorities [4].

One of the most prominent targets of heterocyclic compounds is cancer. Cancer is one of the leading global health challenges, and according to the World Health Organization, cancer-related deaths are expected to reach 12 million by 2030 [5]. Cancer is associated with a wide range of biological processes, each involving multiple subtargets. In this context, protein kinases emerge as significant targets. Protein kinases regulate critical cellular functions such as growth and proliferation in cancerous cells, contributing to the progression of the disease [6]. Additionally, disruptions in the function of these enzymes, which regulate key processes like cell proliferation, signal transduction, and apoptosis, play a crucial role in cancer development [7]. Inhibition of protein kinases by small molecules leads to a suppression of the activities of cancerous cells. Among the kinase families, phosphoinositide 3-kinases (PI3Ks) are of particular importance. These enzymes are involved in regulating cellular functions such as growth, proliferation, motility, and survival, and they play a pivotal role in cancer development [8,9].

**Figure 1:**
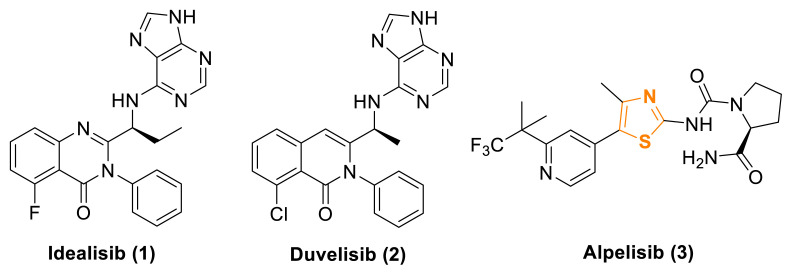
Commercially available PI3K inhibitor drugs.

Currently, only a limited number of PI3K inhibitor drugs are commercially available. One of these, Idelalisib (1), is used in the treatment of chronic lymphocytic leukemia (CLL) [10]. However, its clinical use has been associated with several adverse effects, including immune-mediated hepatotoxicity, diarrhea, fever, fatigue, nausea, cough, pneumonia, abdominal pain, chills, and rash [11]. Another PI3K inhibitor, Duvelisib (2), is typically used as a last-line treatment for diseases such as small lymphocytic lymphoma and follicular lymphoma. However, Duvelisib is also associated with common side effects, including diarrhea, leukopenia, rash, fatigue, fever, and muscle pain, as well as more severe adverse effects, such as neutropenia, pneumonia, and infections [12]. Alpelisib (3) is another PI3K inhibitor used clinically and contains a thiazole ring. However, this drug can also lead to side effects such as high blood sugar, kidney problems, diarrhea, rash, low blood cell counts, liver issues, pancreatitis, vomiting, and hair loss (Figure 1) [13–16].

**Figure 2:**
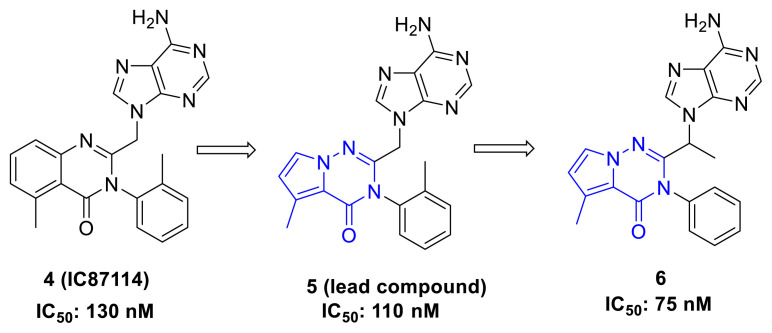
Development of pyrrolotriazinone derivatives as PI3K inhibitors by Erra et al.

The severe side effects observed with these commercially available drugs have accelerated research efforts aimed at developing new PI3K inhibitors. One of the first compounds developed based on the structures of molecules like Idelalisib and Duvelisib is IC87114 (4), which has emerged as a potential inhibitor of the PI3K enzyme, with an IC_50_ value of 130 nM [17]. However, a modified precursor compound (5), which incorporated a pyrrolotriazinone structure, was found to have an improved IC_50_ value of 110 nM [18]. Further optimization of this structure led to compound 6, which demonstrated significantly higher biological activity with an IC_50_ value of 75 nM (Figure 2) [19].

**Figure 3:**
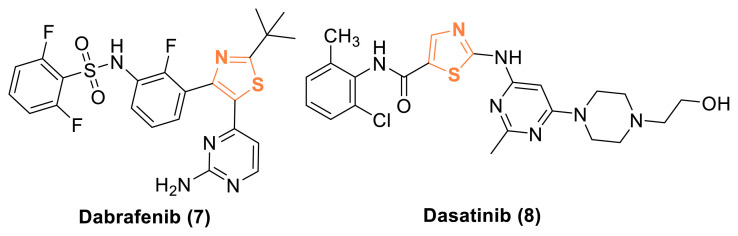
Thiazole-based kinase inhibitors for targeted cancer therapy.

Furthermore, many thiazole derivatives developed in medicinal chemistry have demonstrated potent antitumor or cytotoxic activity, specifically designed to target particular pathways [20,21]. For example, thiazole carboxamide derivatives of tubulysins [22], naphthalene-linked pyrazoline-thiazole hybrids [23], and bis-pyrazolyl-thiazole derivatives [24] have recently been identified as potent antitumor agents, exhibiting cytotoxic activity against various cancer cell lines. Additionally, potent anticancer agents such as Alpelisib (3, Figure 1) [25], Dabrafenib (**7**) [26], and Dasatinib (8) [27] serve as examples of selective anticancer drugs featuring a 1,3-thiazole scaffold, demonstrating kinase inhibitory activity (Figure 3).

**Figure 4:**
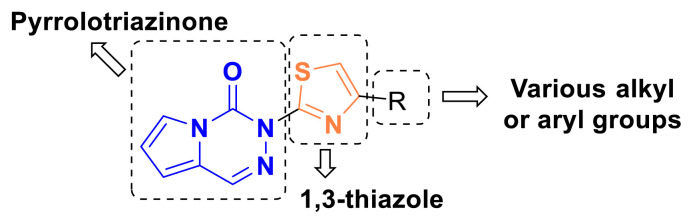
The target scaffold in this work.

Based on all the literature data, the 1,3-thiazole ring scaffold found in various anticancer drugs has been hybridized with the pyrrolotriazinone structure, which possesses PI3K inhibitory properties, into a single molecule in this study (Figure 4). The design of the synthesized compounds aims to investigate their antiproliferative activity in MCF-7, A549, and HepG2 cancer cell lines, as well as their effect on PI3K levels in cancer cells.

## 2. Materials and methods

### 2.1. Materials and instrumentation

Nuclear magnetic resonance (NMR) spectra for proton (^1^H) and carbon-13 (^13^C) were acquired on a Varian-Agilent Inova spectrometer operating at 400 MHz and 100 MHz, respectively, with tetramethylsilane (Me_4_Si) as the internal standard. Liquid chromatography-tandem mass spectrometry (LC-MS/MS) analysis was conducted using a Thermo Scientific Q Exactive MS/MS system (Bremen, Germany) equipped with an electrospray ionization source. Melting points were determined with a Stuart Melting Point (SMP30) (Stafford, UK) apparatus, using open glass capillaries. Column chromatography was performed on silica gel (60 mesh, Silycycle) (Québec, Canada). All commercially available reagents were used as received without further purification.

### 2.2. Chemistry

A series of methods, previously described in the literature, were employed for the synthesis of the target compounds. Initially, commercially available pyrrole (9) was converted to pyrrole-2-carbaldehyde (10) using Vilsmeier-Haack reagents [28]. Subsequently, the N-ethyl formate substitution of pyrrole-2-carbaldehyde (11) was followed by condensation with thiosemicarbazide to yield the corresponding thiosemicarbazone derivative (12) [29].

An appropriate derivative of chloroacetone or 2-bromo-1-(aryl)ethan-1-one (1.05 mmol) was added to a solution of compound 12 (1.0 mmol) in ethanol (10 mL). The mixture was heated under reflux in a sealed tube for 15 min, during which a precipitate formed upon warming. After monitoring the reaction by thin-layer chromatography, the mixture was allowed to cool to room temperature. The reaction mixture was then transferred to 10 mL of an ice-water mixture (1 mL) and stirred at room temperature for 10 min. The resulting solid was filtered through a Buchner funnel, washed several times with cold ethanol (3 mL) and water (5 mL), and then air-dried. The crude products (13–32) were collected after filtration and washing, and subsequently purified by column chromatography on silica gel using an appropriate mobile phase.

### 2.3. MTT assay

MCF-7 (human ER + breast cancer, HTB-22, USA), A549 (human nonsmall cell lung cancer, ATTC CRM-CCL-185, USA), HepG2 (human liver cancer, ATTC HB-8065, USA), and NIH/3T3 (mouse embryonic fibroblast, ATCC, CRL-1658, USA) cells were maintained in DMEM supplemented with 10% fetal bovine serum and 1% penicillin/streptomycin. The cells were maintained under appropriate conditions (37 °C, 5% CO_2_).

The antiproliferative activity of the newly synthesized compounds was measured using the MTT assay as described in a previous study [30]. Briefly, cells were seeded at a density of 6 × 10^3^ cells/well and incubated for 24 h in a 37 °C humidified incubator with 5% CO_2_. Afterwards, the cells were treated with increasing concentrations (1, 2.5, 5, 10, 25, 50, 75, and 100 μM) of compounds and incubated for another 24 h. Moreover, some cells were incubated with doxorubicin (1, 5, 10, 25, and 50 μM) for comparison, while other cells treated only with 1% DMSO was used as the solvent control. After incubation, 20 μL of MTT solution (5 mg/mL in PBS) was added to each well, and the plate was incubated for 4 h under the same conditions. Following this period, the medium was discarded and replaced with DMSO. The absorbance of dissolved formazan crystals was measured using a multiplate reader. The IC_50_ value and the selectivity index of each compound were calculated as previously described [30]. Compounds exhibiting an IC_50_ value below 10 μM and a selectivity index—defined as the ratio of healthy cells to cancer cells exceeding 2—are deemed potential candidates for further studies.

### 2.4. Measurement of PI3K protein levels

The protein levels of PI3K in MCF-7 and HepG2 cells were assessed using enzyme-linked immunosorbent assay (ELISA) kits. The cells were treated with IC_50_ concentrations of the selected compounds for 24 h. After the incubation period, the medium was collected, and PI3K protein levels were measured using ELISA kits according to the manufacturer’s instructions (Cat no: ELK9879, ELK Biotechnology, Denver, USA).

### 2.5. Molecular docking studies

Molecular docking studies were performed using AutoDock 4.2 software (The Scripps Research Institute, San Diego, CA, USA) to investigate the interactions of compounds 21, 25, 26, 27, and the reference structure LASW1579 [31] with the 3D structure of PI3K. The crystal structure of PI3K (PDB ID: 5M6U) was retrieved from the RCSB Protein Data Bank^1^. The molecular structures of the compounds and the reference structure were generated using GaussView 5.0, followed by geometry optimization using density functional theory (DFT) with the B3LYP method and the 6–31G basis set, implemented in the Gaussian 03 package [32]. Docking studies began with the removal of solvent, water molecules, and ligands from the PI3K crystal structure. The docking simulations were centered on the coordinates x: 37.261, y: 16.906, and z: 34.242 for the PI3K active site. A grid box with dimensions 60×60×60 points and a grid spacing of 0.375 Å was used to define the docking region. The lowest energy conformations were selected for further analysis. Water molecules were eliminated using AutoDockTools, and polar hydrogen atoms, Gasteiger partial charges, and Kollman charges were subsequently added to the target structure. Additionally, rotatable bonds of the compounds were defined for flexibility during the docking process. Docking simulations were conducted under validated conditions using AutoDock 4.2. To validate the docking protocol, the native ligand was redocked, and its binding pose was compared to the experimentally determined crystal structure. The Lamarckian genetic algorithm, running 10 iterations with a population size of 300, was employed for the docking simulations. The interactions between PI3K and the docked compounds were analyzed using Discovery Studio Client 4.1 (BIOVIA, Dassault Systèmes, San Diego, CA, USA).

### 2.6. Pre-ADMET calculations

Drug-likeness and absorption, distribution, metabolism, excretion, and toxicity (ADMET) properties of the compounds were determined using online tools. The SMILES of each compound were obtained from SwissADME, and drug-likeness was assessed with SwissADME [33]. Selected ADMET parameters were evaluated using Deep-PK [34].

### 2.7. Statistical analysis

Statistical analysis was conducted on all data, and the results are presented as mean ± SD. The analysis involved three repetitions of the experiments. A two-sided Student’s t-test was employed to compare the data between the control and treated groups, with a p-value of <0.05 deemed statistically significant. Statistical analyses were performed using GraphPad Prism Software version 8 for Windows (GraphPad Software, San Diego, CA, USA).

## 3. Results

### 3.1. Chemistry

**Figure 5:**
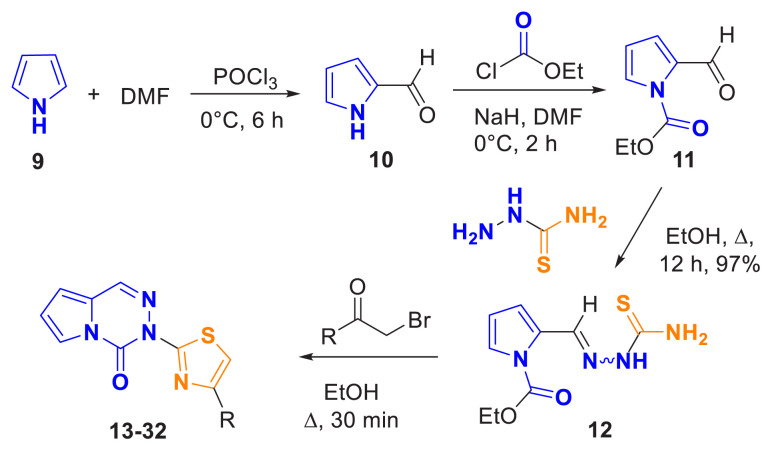
The total synthesis scheme for target compounds.

**Figure 6:**
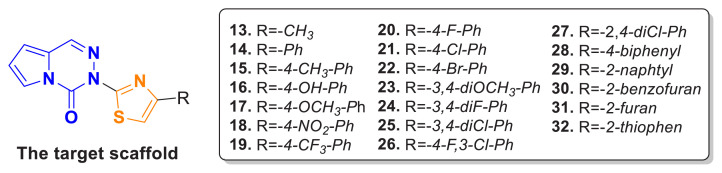
The synthesized target compounds.

The synthetic strategy employed to obtain the target compounds is outlined in Figure 5, while the chemical structures of the synthesized compounds are depicted in Figure 6. NMR analysis and mass data supported the success of the applied route (Supplementary information). It was observed that all aromatic and aliphatic protons in the compounds were as expected in the ^1^H-NMR spectrum, and all ^13^C-NMR spectrum signals were consistent with the type and number of carbons in the compounds. In the design of the target compounds, an alkyl group (-CH_3_ and aryl groups with various substituents were incorporated to investigate the structure-activity relationship in antiproliferative effects. These included electron-donating groups (such as *4*-CH_3_, *4*-OH, *4*-OCH_3,_ and *3,4-*diOCH_3_), electron-withdrawing groups (such as *4*-NO_2_ and *4*-CF_3_), and halogen-substituted phenyl groups (*4*-F, *4*-Cl, *4*-Br, *3,4*-diF, *3,4*-diCl, *3*-Cl, *4*-F, and *2,4*-diCl). Additionally, polyaromatic ring systems (such as biphenyl and naphthyl) and heteroaryl groups (benzofuran, furan, and thiophene) were incorporated.

3-(4-methylthiazol-2-yl)pyrrolo[1,2-*d*][1,2,4]triazin-4(3*H*)-one (13). Brown powder; yield: 81%; mp: 165–166 °C. ^1^H NMR (400 MHz, CDCl_3_) δ 8.32 (s, 1H, Ar-H), 7.91–7.88 (m, 1H, Ar-H), 6.85 (dd, *J* = 1.3 Hz, *J* = 3.7 Hz, 1H, Ar-H), 6.84–6.80 (m, 2H, Ar-H), 2.50 (d, *J* = 1.0 Hz, 3H, -CH_3_). ^13^C NMR (100 MHz, CDCl_3_) δ 149.2, 134.2, 124.3, 119.2, 119.0, 116.8, 111.9, 111.8, 111.6, 17.6. HRMS [M+H]; Calculated for C_10_H_9_N_4_OS: 233.0497, Found: 233.0491.

3-(4-phenylthiazol-2-yl)pyrrolo[1,2-*d*][1,2,4]triazin-4(3*H*)-one (14). Gray powder; yield: 92%; mp: 176–178 °C. ^1^H NMR (400 MHz, CDCl_3_) δ 8.35 (d, *J* = 0.5 Hz, 1H, Ar-H), 7.96–7.93 (m, 2H, Ar-H), 7.92–7.91 (m, 1H, Ar-H), 7.45–7.41 (m, 3H, Ar-H), 7.36–7.32 (m, 1H, Ar-H), 6.87 (dd, *J =* 1.3 Hz, *J =* 3.7 Hz, 1H, Ar-H), 6.85–6.82 (m, 1H, Ar-H). ^13^C NMR (100 MHz, CDCl_3_) δ 156.0, 151.8, 143.6, 134.2, 134.1, 128.6, 128.2, 126.5, 124.3, 119.2, 116.9, 111.7, 111.2. HRMS [M+H]; Calculated for C_15_H_11_N_4_OS: 295.0654, Found: 295.0648.

3-(4-(p-tolyl)thiazol-2-yl)pyrrolo[1,2-*d*][1,2,4]triazin-4(3*H*)-one (15). Dark gray powder; yield: 78%; mp: 205–206 °C. ^1^H NMR (400 MHz, CDCl_3_) δ 8.34 (s, 1H, Ar-H), 7.92–7.88 (m, 1H, Ar-H), 7.86–7.79 (m, AA′BB′ system, 2H, Ar-H), 7.35 (s, 1H, Ar-H), 7.24–7.19 (m, AA′BB′ system, 2H, Ar-H), 6.87–6.81 (m, 2H, Ar-H), 2.37 (s, 3H, Ar-CH_3_). ^13^C NMR (100 MHz, CDCl_3_) δ 157.8, 151.8, 143.6, 138.1, 134.2, 131.4, 129.3, 126.4, 124.3, 119.1, 116.9, 111.7, 110.4, 21.4. HRMS [M+H]; Calculated for C_16_H_13_N_4_OS: 309.0810, Found: 309.0805.

3-(4-(4-hydroxyphenyl)thiazol-2-yl)pyrrolo[1,2-*d*][1,2,4]triazin-4(3*H*)-one (16). Light brown powder; yield: 65%; mp: 150–151 °C. ^1^H NMR (400 MHz, *d_6_*-DMSO) δ 9.64 (s, 1H, -OH), 8.59 (s, 1H, Ar-H), 7.97–7.93 (m, 1H, Ar-H), 7.80–7.76 (m, AA′BB′ system, 2H, Ar-H), 7.74 (s, 1H, Ar-H), 7.00 (dd, *J =* 1.3 Hz, *J =* 3.6 Hz, 1H, Ar-H), 6.90 (dd, *J =* 3.1 Hz, *J =* 3.6 Hz, 1H, Ar-H), 6.84–6.80 (m, AA′BB′ system, 2H, Ar-H). ^13^C NMR (100 MHz, *d_6_*-DMSO) δ 158.2, 157.9, 150.9, 143.7, 134.9, 127.6, 125.8, 125.0, 119.3, 117.2, 115.9, 112.2, 110.4. HRMS [M+H]; Calculated for C_15_H_11_N_4_O_2_S: 311.0603, Found: 311.0597.

3-(4-(4-methoxyphenyl)thiazol-2-yl)pyrrolo[1,2-*d*][1,2,4]triazin-4(3*H*)-one (17). Gray powder; yield: 90%; mp: 150–152 °C. ^1^H NMR (400 MHz, *d_6_*-DMSO) δ 8.59 (s, 1H, Ar-H), 7.97–7.93 (m, 1H, Ar-H), 7.92–7.87 (m, AA′BB′ system, 2H, Ar-H), 7.83 (s, 1H, Ar-H), 7.01–6.98 (m, 3H, Ar-H), 6.90–6.89 (m, 1H, Ar-H), 3.78 (s, 3H, -OCH_3_). ^13^C NMR (100 MHz, *d_6_*-DMSO) 164.4, 163.1, 155.3, 148.5, 139.7, 132.4, 132.0, 129.7, 124.1, 121.9, 119.3, 117.0, 115.9, 60.4. HRMS [M+H]; Calculated for C_16_H_13_N_4_O_2_S: 325.0759, Found: 325.0753.

3-(4-(4-nitrophenyl)thiazol-2-yl)pyrrolo[1,2-*d*][1,2,4]triazin-4(3*H*)-one (18). Yellow powder; yield: 70%; mp: 241–242 °C. ^1^H NMR (400 MHz, *d_6_*-DMSO) δ 8.64 (bs, 1H, Ar-H), 8.39–8.32 (m, 3H, Ar-H), 8.26–8.24 (m, AA′BB′ system, 2H, Ar-H), 8.01–7.96 (m, 1H, Ar-H),7.04 (dd, *J =* 1.3 Hz, *J =* 3.6 Hz, 1H, Ar-H), 6.95–6.90 (m, 1H, Ar-H). ^13^C NMR (100 MHz, *d_6_*-DMSO) δ 159.1, 148.3, 147.2, 143.9, 140.4, 135.2, 127.2, 125.0, 124.8, 119.6, 117.3, 117.2, 112.5. HRMS [M+H]; Calculated for C_15_H_10_N_5_O_3_S: 340.0504, Found: 340.0498.

3-(4-(4-(trifluoromethyl)phenyl)thiazol-2-yl)pyrrolo[1,2-*d*][1,2,4]triazin-4(3*H*)-one (19). Dark gray powder; yield: 95%; mp: 203–204 °C. ^1^H NMR (400 MHz, CDCl_3_) δ 8.36 (s, 1H, Ar-H), 8.06 (m, AA′BB′ system, 2H, Ar-H), 7.94–7.91 (m, 1H, Ar-H), 7.70–7.65 (m, AA′BB′ system, 2H, Ar-H), 7.52 (s, 1H, Ar-H), 6.89 (dd, *J =* 1.3 Hz, *J =* 3.7 Hz, 1H, Ar-H), 6.87–6.83 (m, 1H, Ar-H). ^13^C NMR (100 MHz, CDCl_3_) δ 158.4, 150.2, 143.6, 137.3, 134.4, 130.1, 126.6, 125.6 (q, *J =* 3.8 Hz), 124.3, 122.8, 119.3, 117.0, 113.0, 112.0. HRMS [M+H]; Calculated for C_16_H_10_F_3_N_4_OS: 363.0527, Found: 363.0521.

3-(4-(4-fluorophenyl)thiazol-2-yl)pyrrolo[1,2-*d*][1,2,4]triazin-4(3*H*)-one (20). Light gray powder; yield: 86%; mp: 199–200 °C. ^1^H NMR (400 MHz, CDCl_3_) δ 8.27 (s, 1H, Ar-H), 7.88–7.82 (m, 3H, Ar-H), 7.27 (s, 1H, Ar-H), 7.07–7.01 (m, 2H, Ar-H), 6.82–6.75 (m, 2H, Ar-H). ^13^C NMR (100 MHz, CDCl_3_) δ 162.82 (d, *J =* 247.4 Hz), 158.1, 150.9, 143.6, 134.3, 130.5, 128.3, 124.4, 119.3, 116.9, 115.7, 115.5, 111.8, 110.9. HRMS [M+H]; Calculated for C_16_H_10_FN_4_OS: 313.0559, Found: '313.0553'.

3-(4-(4-chlorophenyl)thiazol-2-yl)pyrrolo[1,2-*d*][1,2,4]triazin-4(3*H*)-one (21). Gray powder; yield: 93%; mp: 178–181 °C. ^1^H NMR (400 MHz, CDCl_3_) δ 8.34 (d, *J* = 0.5 Hz, 1H, Ar-H), 7.91–7.90 (m, 1H, Ar-H), 7.90–7.86 (m, AA′BB′ system, 2H, Ar-H), 7.40–7.37 (m, 3H, Ar-H), 6.87 (dd, *J =* 1.3 Hz, *J =* 3.7 Hz, 1H, Ar-H), 6.85–6.82 (m, 1H, Ar-H). ^13^C NMR (100 MHz, CDCl_3_) δ 158.2, 150.6, 134.3, 134.0, 132.6, 129.7, 128.8, 127.7, 124.3, 119.2, 116.9, 111.9, 111.5. HRMS [M+H]; Calculated for C_15_H_10_ClN_4_OS: 329.0264, Found: 329.0258.

3-(4-(4-bromophenyl)thiazol-2-yl)pyrrolo[1,2-*d*][1,2,4]triazin-4(3*H*)-one (22). Brown powder; yield: 88%; mp: 207–208 °C. ^1^H NMR (400 MHz, CDCl_3_) δ 8.62 (d, *J =* 0.6 Hz, 1H, Ar-H), 8.08 (s, 1H, Ar-H), 7.97 (ddd, *J =* 0.6 Hz, *J =* 1.3 Hz, *J =* 3.0 Hz, 1H, Ar-H), 7.96–7.92 (m, AA′BB′ system, 2H, Ar-H), 7.68–7.64 (m, AA′BB′ system, 2H, Ar-H), 7.03 (dd, *J =* 1.3 Hz, *J =* 3.7 Hz, 1H, Ar-H), 6.92 (d, *J =* 3.0 Hz, *J =* 3.7 Hz, 1H, Ar-H). ^13^C NMR (100 MHz, CDCl_3_) δ 158.7, 149.4, 143.8, 135.0, 133.7, 132.2, 128.3, 125.0, 121.7, 119.5, 117.3, 113.8, 112.4. HRMS [M+H]; Calculated for C_15_H_10_BrN_4_OS: 372.9759, Found: 372.9753.

3-(4-(3,4-dimethoxyphenyl)thiazol-2-yl)pyrrolo[1,2-*d*][1,2,4]triazin-4(3*H*)-one (23). Gray powder; yield: 71%; mp: 217–218 °C. ^1^H NMR (400 MHz, CDCl_3_) δ 8.33 (d, *J =*0.4 Hz, 1H, Ar-H), 7.94–7.88 (m, 1H, Ar-H), 7.52–7.45 (m, 2H, Ar-H), 7.30 (s, 1H, Ar-H), 6.92 (d, *J =* 8.3 Hz, 1H, Ar-H), 6.87 (dd, *J =* 1.3 Hz, *J =* 3.7 Hz, 1H, Ar-H), 6.84 (dd, *J =* 3.0 Hz, *J =* 3.7 Hz, 1H, Ar-H), 3.99 (s, 3H, -OCH_3_), 3.93 (s, 3H, -OCH_3_). ^13^C NMR (100 MHz, CDCl_3_) δ 157.9, 151.8, 149.3, 149.0, 143.6, 134.1, 127.5, 124.4, 119.2, 119.1, 116.9, 111.7, 111.2, 110.0, 109.8, 56.0, 55.9. HRMS [M+H]; Calculated for C_17_H_15_N_4_O_3_S: 355.0865, Found: 355.0859.

3-(4-(3,4-difluorophenyl)thiazol-2-yl)pyrrolo[1,2-*d*][1,2,4]triazin-4(3*H*)-one (24). Dark brown powder; yield: 79%; mp: 193–194°C. ^1^H NMR (400 MHz, CDCl_3_) δ 8.34 (s, 1H, Ar-H), 7.93–7.86 (m, 1H, Ar-H), 7.82–7.73 (m, 1H, Ar-H), 7.69–7.61 (m, 1H, Ar-H), 7.35 (s, 1H, Ar-H), 7.20 (dt, *J =* 8.3 Hz, *J =* 10.1 Hz, 1H, Ar-H), 6.88 (dd, *J =* 1.3 Hz, *J =* 3.7 Hz, 1H, Ar-H), 6.85–6.82 (m, 1H, Ar-H). ^13^C NMR (100 MHz, CDCl_3_) δ 158.3, 151.7 (dd, *J =* 12.4 Hz, *J =* 17.0 Hz), 149.7, 149.2 (dd, *J =*12.4 Hz, *J =* 17.0 Hz), 143.6, 134.4, 131.4, (dd, *J =* 3.7 Hz, *J =* 6.3 Hz), 124.3, 122.4 (dd, *J =*3.7 Hz, *J =* 6.3 Hz), 119.3, 117.5 (d, *J =* 17.0 Hz), 117.0, 115.6 (d, *J =* 17.0 Hz), 111.9, 111.7. HRMS [M+H]; Calculated for C_15_H_9_F_2_N_4_OS: 331.0465, Found: 331.0459.

3-(4-(3,4-dichlorophenyl)thiazol-2-yl)pyrrolo[1,2-*d*][1,2,4]triazin-4(3*H*)-one (25). Gray powder; yield: 89%; mp: 200–201 °C. ^1^H NMR (400 MHz, CDCl_3_) δ 8.34 (s, 1H, Ar-H), 8.04 (d, *J =* 2.1 Hz, 1H, Ar-H), 7.91–7.88 (m, 1H, Ar-H), 7.75 (dd, *J =* 2.1 Hz, *J =* 8.4 Hz, 1H, Ar-H), 7.46 (d, *J =* 8.4 Hz, 1H, Ar-H), 7.41 (s, 1H, Ar-H), 6.88 (dd, *J =* 1.3 Hz, *J =* 3.7 Hz, 1H, Ar-H), 6.85–6.81 (m, 1H, Ar-H). ^13^C NMR (100 MHz, CDCl_3_) δ 158.3, 149.3, 143.6, 134.4, 134.0, 132.9, 132.0, 130.4, 128.2, 125.4, 124.2, 119.2, 117.0, 112.5, 112.0. HRMS [M+H]; Calculated for C_15_H_9_Cl_2_N_4_OS: 362.9874, Found: 362.9868.

3-(4-(3-chloro-4-fluorophenyl)thiazol-2-yl)pyrrolo[1,2-*d*][1,2,4]triazin-4(3*H*)-one (26). Light green powder; yield: 91%; mp: 183–184 °C. ^1^H NMR (400 MHz, CDCl_3_) δ 8.27 (bs, 1H, Ar-H), 7.93 (dd, *J =* 2.2 Hz, *J =* 7.1 Hz, 1H, Ar-H), 7.86–7.79 (m, 1H, Ar-H), 7.75–7.66 (m, 1H, Ar-H), 7.29 (s, 1H, Ar-H), 7.10 (t, *J =* 8.7 Hz, 1H, Ar-H), 6.83–6.75 (m, 2H, Ar-H). ^13^C NMR (100 MHz, CDCl_3_) δ 158.3, 158.0 (d, *J =* 250.1), 149.5, 143.6, 134.4, 131.5 (d, *J =* 4.0 Hz), 128.7, 126.2 (d, *J =* 7.3 Hz), 124.3, 121.4 (d, *J =* 18.1 Hz), 119.3, 117.0, 116.7 (d, *J =* 21.3 Hz), 112.0, 111.7. HRMS [M+H]; Calculated for C_15_H_9_ClFN_4_OS: 347.0170, Found: 347.0164.

3-(4-(2,4-dichlorophenyl)thiazol-2-yl)pyrrolo[1,2-*d*][1,2,4]triazin-4(3*H*)-one (27). White powder; yield: 85%; mp: 181–182 °C. ^1^H NMR (400 MHz, CDCl_3_) δ 8.26 (bs, 1H, Ar-H), 7.88 (d, *J =* 8.4 Hz, 1H, Ar-H), 7.85 (ddd, *J =* 0.6 Hz, *J =* 1.3 Hz, *J =* 3.0 Hz, 1H, Ar-H), 7.67 (s, 1H, Ar-H), 7.41 (d, *J =* 2.1 Hz, 1H, Ar-H), 7.26 (dd, *J =* 2.1 Hz, *J =* 8.4 Hz, 1H, Ar-H), 6.81 (dd, *J =* 1.3 Hz, *J =* 3.7 Hz, 1H, Ar-H), 6.77 (dd, *J =* 3.0 Hz, *J =* 3.7 Hz, 1H, Ar-H). ^13^C NMR (100 MHz, CDCl_3_) δ 152.4, 142.3, 138.9, 129.6, 129.4, 127.9, 127.8, 126.9, 125.3, 122.6, 119.6, 114.5, 112.2, 112.0, 107.2. HRMS [M+H]; Calculated for C_15_H_9_Cl_2_N_4_OS: 362.9874, Found: 362.9869.

3-(4-([1,1′-biphenyl]-4-yl)thiazol-2-yl)pyrrolo[1,2-*d*][1,2,4]triazin-4(3*H*)-one (28). Light brown powder; yield: 95%; mp: 226–228 °C. ^1^H NMR (400 MHz, CDCl_3_) δ 8.62 (d, *J =* 0.6 Hz, 1H, Ar-H), 8.09–8.05 (m, 3H, Ar-H), 7.98–7.96 (m, 1H, Ar-H), 7.78–7.75 (m, AA′BB′ system, 2H, Ar-H), 7.74–7.70 (m, AA′BB′ system, 2H, Ar-H), 7.50–7.45 (m, 2H, Ar-H), 7.39–7.35 (m, 1H, Ar-H), 7.02 (dd, *J =* 1.3 Hz, *J =* 3.7 Hz, 1H, Ar-H), 6.91 (dd, *J =* 3.0 Hz, *J =* 3.7 Hz, 1H, Ar-H). ^13^C NMR (100 MHz, CDCl_3_) δ 158.6, 150.2, 143.8, 140.1, 140.0, 135.0, 133.5, 129.4, 128.0, 127.5, 127.0, 126.9, 125.0, 119.4, 117.2, 113.2, 112.3. HRMS [M+H]; Calculated for C_21_H_15_N_4_OS: 371.0967, Found: 371.0961.

3-(4-(naphthalen-2-yl)thiazol-2-yl)pyrrolo[1,2-*d*][1,2,4]triazin-4(3*H*)-one (29). Light gray powder; yield: 96%; mp: 214–215 °C. ^1^H NMR (400 MHz, CDCl_3_) δ 8.53–8.50 (m, 1H, Ar-H), 8.40–8.38 (m, 1H, Ar-H), 8.00 (dd, *J =* 1.8 Hz, *J =* 8.6 Hz, 1H, Ar-H), 7.96–7.92 (m, 2H, Ar-H), 7.89 (d, *J =* 8.6 Hz, 1H, Ar-H), 7.86–7.82 (m, 1H, Ar-H), 7.54 (s, 1H, Ar-H), 7.51–7.46 (m, 2H, Ar-H), 6.89 (dd, *J =* 1.3 Hz, *J =* 3.7 Hz, 1H, Ar-H), 6.86–6.83 (m, 1H, Ar-H). ^13^C NMR (100 MHz, CDCl_3_) δ 158.1, 151.7, 143.7, 134.3, 133.5, 133.2, 131.4, 128.5, 128.3, 127.7, 126.3, 126.1, 125.6, 124.3, 124.2, 119.2, 116.9, 111.8, 111.7. HRMS [M+H]; Calculated for C_19_H_13_N_4_OS: 345.0810, Found: 345.0804.

3-(4-(benzofuran-2-yl)thiazol-2-yl)pyrrolo[1,2-*d*][1,2,4]triazin-4(3*H*)-one (30). Dark brown powder; yield: 81%; mp: 231–232 °C. ^1^H NMR (400 MHz, CDCl_3_) δ 8.36 (s, 1H, Ar-H), 7.92 (d, *J =* 2.3 Hz, 1H, Ar-H), 7.67–7.58 (m, 2H, Ar-H), 7.52 (d, *J =* 8.1 Hz, 1H, Ar-H), 7.30–7.22 (m, 3H, Ar-H), 6.93–6.80 (m, 2H, Ar-H). ^13^C NMR (100 MHz, CDCl_3_) δ 158.6, 154.9, 151.3, 143.6, 143.2, 134.4, 128.9, 124.7, 124.3, 123.1, 121.5, 119.3, 117.0, 113.1, 112.0, 111.2, 103.9. HRMS [M+H]; Calculated for C_17_H_11_N_4_O_2_S: 335.0603, Found: 335.0597.

3-(4-(furan-2-yl)thiazol-2-yl)pyrrolo[1,2-*d*][1,2,4]triazin-4(3*H*)-one (31). White powder; yield: 77%; mp: 236–237 °C. ^1^H NMR (400 MHz, CDCl_3_) δ 8.27 (d, *J =* 0.5 Hz, 1H, Ar-H), 7.84 (ddd, *J =* 0.7 Hz, *J =* 1.3 Hz, *J =* 3.0 Hz, 1H, Ar-H), 7.39 (dd, *J =* 0.7 Hz, *J =* 1.8 Hz, 1H, Ar-H), 7.29 (s, 1H, Ar-H), 6.84 (dd, *J =* 0.5 Hz, *J =* 3.4 Hz, 1H, Ar-H), 6.80 (dd, *J =* 1.3 Hz, *J =* 3.7 Hz, 1H, Ar-H), 6.77 (dd, *J =* 3.0 Hz, *J =* 3.7 Hz, 1H, Ar-H), 6.42 (d, *J =* 1.8 Hz, *J =* 3.4 Hz, 1H, Ar-H). ^13^C NMR (100 MHz, CDCl_3_) δ 158.3, 149.8, 143.6, 143.5, 142.3, 134.3, 124.3, 119.3, 116.9, 111.9, 111.5, 110.4, 107.5. HRMS [M+H]; Calculated for C_13_H_9_N_4_O_2_S: 285.0446, Found: 285.0440.

3-(4-(thiophen-2-yl)thiazol-2-yl)pyrrolo[1,2-*d*][1,2,4]triazin-4(3*H*)-one (32). Dark brown powder; yield: 90%; mp: 211–213 °C. ^1^H NMR (400 MHz, CDCl_3_) δ 8.34 (d, *J =* 0.5 Hz, 1H, Ar-H), 7.91–7.89 (m, 1H, Ar-H), 7.52 (dd, *J =* 1.2 Hz, *J =* 3.6 Hz, 1H, Ar-H), 7.29 (dd, *J =* 1.2 Hz, *J =* 5.1 Hz, 1H, Ar-H), 7.26 (s, 1H, Ar-H), 7.07 (dd, *J =* 3.6 Hz, *J =* 5.1 Hz, 1H, Ar-H), 6.87 (dd, *J =* 1.3 Hz, *J =* 3.7 Hz, 1H, Ar-H), 6.84–6.82 (m, 1H, Ar-H). ^13^C NMR (100 MHz, CDCl_3_) δ 158.0, 146.4, 138.0, 134.3, 127.7, 125.3, 124.7, 124.3, 119.2, 116.9, 111.8, 110.2, 110.1. HRMS [M+H]; Calculated for C_13_H_9_N_4_OS_2_: 301.0218, Found: 301.0212.

### 3.2. Biological activity and structure-activity relationship

The antiproliferative effect of all newly synthesized thiazole-integrated pyrrolotriazinone derivatives were assessed against cancer cell lines (MCF-7, A549, and HepG2) as well as a healthy cell line (NIH/3T3) using the MTT assay. The IC_50_ values were determined through the nonlinear regression algorithm for log (inhibitor) versus normalized response-variable slope, as implemented in GraphPad [Y = 100/(1 + 10^X−LogIC50^), where X represents the logarithm of the inhibitor concentration and Y denotes the response]. Additionally, Selectivity Indices (SI: IC_50_ of healthy cells/IC_50_ of cancer cells) were calculated. The results are presented in Table 1.

**Figure 7:**
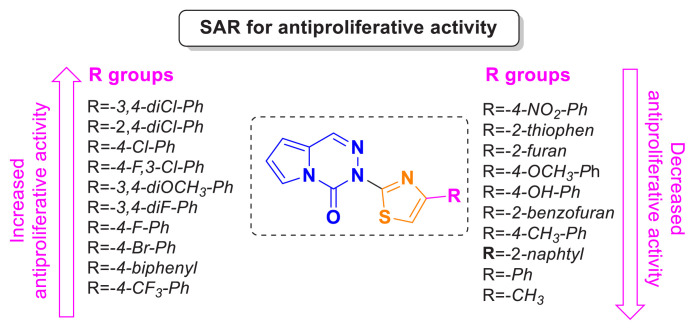
SAR of the synthesized compounds on antiproliferative activity.

Based on the antiproliferative activity results, significant structure-activity relationships can be established for the synthesized compounds (Figure 7). When evaluating the tested cancer cell lines overall, compound 13, which carries a -CH_3_ substitution on the alkyl group, shows the lowest antiproliferative activity. When the methyl group is replaced by a phenyl group in compound 14, the activity increases slightly, and electron-donating groups with progressively higher electron density, such as *4*-CH_3_, *4*-OH, and *4*-OCH_3_ (compounds 15, 16, and 17, respectively), demonstrate a corresponding increase in antiproliferative activity. This hypothesis is further supported by the satisfactory antiproliferative activity of compound 23, which contains a *3,4*-dimethoxy group. Moreover, compounds 18 and 19, which contain electron-withdrawing groups (*4*-NO_2_ and *4*-CF_3_, respectively), exhibit generally higher activity compared to electron-donating groups, although their activity remains lower than that of compound 23 with the dimethoxy group. This suggests a delicate balance between the influence of electron-donating and electron-withdrawing groups on antiproliferative activity. Compound 19, bearing the *4*-CF_3_ group, shows a significantly higher antiproliferative activity than compound 18 with the *4*-NO_2_ group, highlighting the importance of halogen substitution in enhancing antiproliferative activity.

In line with this, compounds 20, 21, and 22, which carry *4*-F, *4*-Cl, and *4*-Br substitutions, respectively, show that the compound with the *4*-Cl substituent (compound 21) exhibits the highest activity. This suggests that the presence of a bulky halogen group, such as -Br, may reduce antiproliferative activity due to a decrease in its electron-withdrawing effect. Additionally, among the compounds with *3,4*-diF, *3,4*-diCl, and *3*-Cl,*4*-F structures (compounds 24, 25, and 26, respectively), compound 25 demonstrates the highest antiproliferative activity. This finding underscores the critical role of a -Cl substitution at the *4*-position in enhancing antiproliferative activity. Consequently, the *2,4*-diCl-substituted compound (27) exhibits antiproliferative activity comparable to compound 25 with a *3,4*-diCl substitution.

Furthermore, compounds containing polyaromatic groups, such as biphenyl and naphthyl (compounds 28 and 29), show reduced antiproliferative activity. In heteroaromatic systems, the compound containing a benzofuran structure (compound 30) is less active compared to the compound with a furan structure (compound 31), whereas antiproliferative activity is relatively enhanced in the thiophene-substituted compound 32.

In conclusion, these data suggest that electron-donating groups play a significant role in the activity, while polyaromatic and heteroaromatic groups contribute less to the activity. Among electron-withdrawing groups, halogen-containing groups notably enhance activity. Specifically, the presence of a *4*-Cl substituent on the phenyl group is critical for maximizing antiproliferative activity. Based on these findings, compounds 21, 25, 26, and 27, each containing a *4*-Cl substituent, were selected for the analysis of PI3K protein levels in MCF-7 and HepG2 cells.

### 3.3. PI3K protein levels

**Figure 8:**
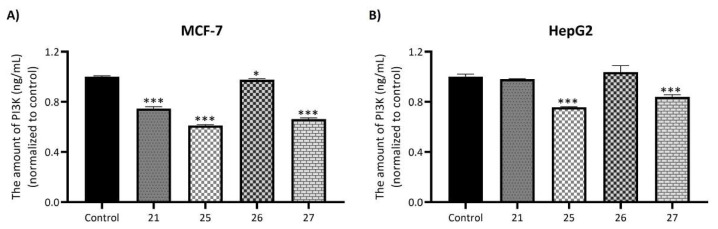
PI3K protein levels in MCF-7 (A) and HepG2 (B) cells. Cells treated with 1% DMSO were accepted as a control. *p ≤ 0.05; ***p ≤ 0.001

The protein levels of PI3K were assessed in the cells following treatment with IC_50_ concentrations of compounds 21, 25, 26, and 27. In MCF-7 cells, all compounds significantly reduced the protein levels of PI3K protein compared to the control in the presence of all four compounds (Figure 8A). In HepG2 cells, only compounds 25 and 27 markedly diminished PI3K protein levels compared to the control (Figure 8B).

### 3.4. In silico studies

#### 3.4.1. Molecular docking studies

Molecular docking studies were performed to test the binding affinity and binding mode of compound 21, 25, 26, and 27 structures, whose activity against PI3K levels was tested in MCF-7 and HepG2 cancer cells. LASW1579, an orally available PI3K inhibitor featuring a pyrrolotriazinone scaffold identified as a coligand in the PI3K crystal structure, was used to pinpoint the active site in molecular docking studies and served as a control for comparison in the docking analysis of the target compounds. Molecular docking studies of compounds 21, 25, 26, and 27 with PI3K were conducted using Autodock 4.2 software, targeting the active site where the coligand is bound. The results are summarized in Table 2.

In the molecular docking studies, the Cluster root-mean-square deviation (RMSD) value was calculated to confirm the binding of the compounds to the target active site of PI3K. For the docking results to be considered valid, the RMSD value should fall within the range of 0–2 Å, and the calculated RMSD values for the compounds in this study were found to be within this acceptable range. With respect to the PI3K active site, compounds 21 (−7.98 kcal/mol) and 26 (−8.05 kcal/mol) exhibit binding affinities comparable to the reference structure LASW1579 (−8.01 kcal/mol). In contrast, compounds 25 (−8.53 kcal/mol) and 27 (−8.30 kcal/mol) demonstrate higher binding affinities, which correlates with the PI3K expression levels observed in cancer cells in vitro for these compounds.

**Figure 9:**
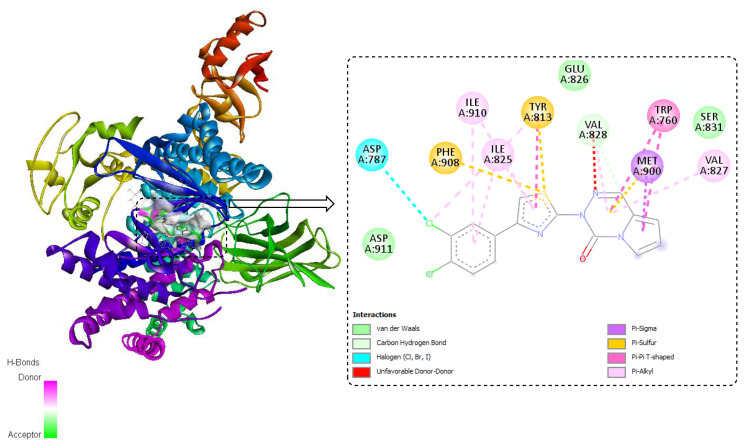
3D and 2D ligand-protein interactions of PI3K active site with compound **25**.

The coligand LASW1579 interacted with several residues within the PI3K active site, including Met752, Pro758, Ile777, Ile825, Glu826, Val827, Val828, Met900, and Ile910. Among the compounds tested, compound 26 showed interaction with only two residues (Val287 and Val828) that overlap with the reference compound, and did not exhibit any significant effect on PI3K levels in vitro. Compound 21, which interacted with three common residues (Ile825, Val827, and Met900) in comparison to the reference compound, did not alter PI3K levels in the HepG2 cancer cell line but did reduce PI3K levels in the MCF-7 cancer cell line. Compound 27, which shared interactions with Met752, Pro758, Ile825, Val828, and Ile910 with the reference compound, reduced PI3K levels in both cancer cell lines in vitro. Finally, compound 25, which demonstrated the most potent inhibitory activity in the in vitro assays, was positioned compatibly within the PI3K active site, interacting with Ile825, Val827, Val828, Met900, and Ile910, similar to the reference compound LASW1579 (Figure 9).

In conclusion, the binding affinities of compounds 21, 25, 26, and 27, which were evaluated for their effects on PI3K protein levels in HepG2 and MCF-7 cancer cells, were assessed in relation to the relevant biological target using a reference scaffold with a pyrrolotriazinone structure designed for oral administration. The molecular docking results are consistent with the in vitro findings, supporting the potential of these compounds as PI3K inhibitors. Therefore, these compounds hold promise as anticancer drug candidates, likely exerting their effects through PI3K-mediated apoptosis. These findings may contribute to the development of novel drug candidates for cancer therapy.

#### 3.4.2. Pre-ADMET calculations for compounds 21, 25, 26, and 27

Predicting ADMET properties with in silico tools aids the drug development process. Therefore, drug-likeness (based on Lipinski’s rule of five) and selected ADMET parameters were evaluated using SwissADME and Deep-PK, respectively. The results are presented in Table 3. The analysis revealed that all four compounds comply with Lipinski’s rule of five and are absorbable from the human intestine (HIA: Human Intestinal Absorption). Molecules with an absorption rate below 30% are considered poorly absorbed. These compounds have high plasma protein binding (PPB), ranging from 96.1% to 99.87%. Additionally, all four compounds show potential as inhibitors of CYP1A2. In terms of clearance, the predicted values suggest moderate to high clearance rates, with molecule 21 exhibiting the highest clearance at 6.66 Log mL/min/kg and molecule 26 showing the lowest at 5.59 Log mL/min/kg. This variation implies that molecule 21 may be eliminated more rapidly, potentially requiring more frequent dosing or specialized formulations to maintain therapeutic levels. Conversely, molecule 26, with its lower clearance rate, may possess a longer half-life and a greater risk of accumulation. Predicted toxicity results indicate that all four compounds are moderately toxic to the liver but safe regarding cardiotoxicity (hERG blockers) and carcinogenicity (Table 3).

## 4. Conclusion

In this study, a series of thiazole-integrated pyrrolotriazinone derivatives (compounds 13–32) were synthesized and evaluated for their potential as apoptosis-inducing agents in cancer therapy. The compounds demonstrated significant antiproliferative activity, particularly against MCF-7 and HepG2 cancer cell lines. Structure-activity relationship (SAR) analysis identified that derivatives containing a phenyl-chloro substituent exhibited enhanced cytotoxic effects. Among the compounds tested, 21, 25, 26, and 27 were found to be the most potent, with compounds 25 and 27 showing a significant reduction in PI3K protein levels in MCF-7 and HepG2 cells. Molecular docking studies further validated their potential as PI3K inhibitors. In silico assessments of drug-likeness and pre-ADMET properties indicated that these compounds possess favorable pharmacokinetic profiles, supporting their further development as therapeutic agents. Overall, these findings underscore the therapeutic potential of thiazole-integrated pyrrolotriazinone derivatives and provide essential insights into the design of novel anticancer agents targeting key signaling pathways such as PI3K. Future research should focus on optimizing these compounds and exploring their clinical potential in cancer treatment.

## Supplementary Information



## Figures and Tables

**Table 1:** IC_50_ values and selectivity indices of the compounds against MCF-7, A549, HepG2, and NIH/3T3 cells.

Compounds	IC_50_ (μM)				Selectivity Index (SI)		
	MCF-7	A549	HepG2	NIH/3T3	MCF-7	A549	HepG2
**13**	182.18±0.26	195.72±1.63	138.95±0.41	183.77 ± 0.5	1.01	0.94	1.32
**14**	165.12±1.13	127.06±0.42	153.27±0.16	159.19± 0.2	0.96	1.25	1.04
**15**	140.59±0.57	169.02±1.05	168.01±0.73	142.14± 1.2	1.01	0.84	0.85
**16**	129.71±0.69	108.9±0.84	141.18±0.29	143.26± 1.0	1.10	1.32	1.02
**17**	117.23±1.18	109.15±1.04	129.28±0.93	157.44± 0.9	1.34	1.44	1.22
**18**	79.19±0.61	109.17±1.94	102.61±1.22	164.43± 0.8	2.08	1.51	1.60
**19**	61.76±1.42	79.51±1.13	52.83±0.79	148.81± 0.4	2.41	1.87	2.82
**20**	39.81±0.54	44.54±0.51	69.74±1.26	196.21± 1.6	4.93	4.41	2.81
**21**	**11.19±0.12**	**39.16±1.41**	**10.23±0.95**	**106.4± 0.84**	**9.51**	**2.72**	**10.40**
**22**	55.63±1.05	71.19±0.74	51.85±0.23	93.46±0.74	1.68	1.31	1.80
**23**	15.21±0.183	43.26±1.09	17.19±0.81	83.44± 0.15	5.49	2.85	4.85
**24**	33.19±0.49	45.23±0.41	25.29±1.06	156.61± 0.5	4.72	3.46	6.19
**25**	**8.1±0.17**	**38.7±0.40**	**7.21±058**	**109.81± 0.6**	**13.60**	**2.84**	**15.20**
**26**	**14.2±0.42**	**25.16±0.39**	**15.09±0.61**	**139.19± 0.2**	**8.59**	**5.53**	**9.22**
**27**	**9.23±1.09**	**37.21±1.15**	**6.18±0.63**	**105.42± 0.3**	**10.30**	**2.83**	**14.01**
**28**	63.17±0.35	78.23±0.05	44.8±1.06	176.27± 0.4	2.79	2.25	3.93
**29**	161.16±1.05	140.17±1.25	132.17±0.63	166.21±0.32	1.03	1.19	1.26
**30**	129.15±0.91	134.39±0.67	107.15±1.31	203.17± 1.4	1.57	1.51	1.90
**31**	91.15±1.06	104.04±0.57	103.26±0.43	174.03± 1.1	1.91	1.67	1.69
**32**	83.71±0.81	121.16±0.26	101.72±1.16	203.26±1.1	2.43	1.68	2.00
**Dox**	1.34±0.07	0.61±0.12	1.27±0.24	3.56±0.02	2.6	5.83	2.8

**Table 2:** Docking scores of compounds 21, 25, 26, 27 and coligand against PI3K.

Compounds	PI3K (PDB ID: 5M6U)			
	Cluster RMSD (Å)	Binding affinity (kcal/mol)	Inhibitory potential (μM)	Interacted amino acid residues
**21**	0.64	−7.98	1.41	Trp760, Tyr813, Ile825, Val827, Leu829, Arg830, Ser831, Met900
**25**	0.70	−8.53	0.56	Trp760, Asp787, Tyr813, Ile825, Val827, Val828, Met900, Phe908, Ile910
**26**	0.57	−8.05	1.25	Trp760, Tyr813, Val827, Val828, Leu829, Ser831
**27**	0.35	−8.30	0.83	Met752, Pro758, Leu759, Trp760, Ile777, Ile825, Val828, Ile910
**LASW1579**	0.65	−8.01	1.37	Met752, Pro758, Ile777, Ile825, Glu826, Val827, Val828, Met900, Ile910

**Table 3:** Drug-likeness and selected ADMET parameters of compounds 21, 25, 26, and 27.

Drug-likeness	21	25	26	27
Lipinski’s rule of five	Yes	Yes	Yes	Yes
**ADMET**				
HIA	Absorbed Confidence: 0.998	Absorbed Confidence: 0.999	Absorbed Confidence: 0.998	Absorbed Confidence: 0.999
PPB (%)	96.13	99.45	96.1	99.87
Metabolism	CYP1A2 inhibitor confidence: 0.986	CYP1A2 inhibitor confidence: 0.978	CYP1A2 inhibitor confidence: 0.96	CYP1A2 inhibitor confidence: 0.97
Clearance Log (mL/min/kg)	6.66	5.82	5.59	5.68
Liver injury (DILI)	Toxic (0.764)	Toxic (0.705)	Toxic (0.723)	Toxic (0.696)
hERG blockers	Safe (0.034)	Safe (0.078)	Safe (0.076)	Safe (0.084)
Carcinogenesis	Safe (0.334)	Safe (0.327)	Safe (0.313)	Safe (0.326)

## Footnotes

1 RSCB (2024). Protein Data Bank [online]. Website https://www.rcsb.org/ [accessed 04 November 2024].
