# Supplementary material for: Synthesis of thiazole-integrated pyrrolotriazinones: evaluations of cytotoxicity and effects on PI3K levels in cancer cells
Source: Turk J Chem. 2025 Jan 20;49(2):215–27. doi: 10.55730/1300-0527.3723 (PMC12068666; doi:10.55730/1300-0527.3723)
Supplement: Supplementary file 1 [file Supp-tjc-49-02-215.doc]

**Synthesis of Thiazole-Integrated Pyrrolotriazinones: Evaluations of Cytotoxicity and Effects on PI3K Levels in Cancer Cells**

Eylem KUZU^1^, Ege ARZUK^2^, Fuat KARAKUŞ^3^, Burak KUZU^4^, Hasan GENÇ^5^*

^1^Chemistry Section, Institute of Natural and Applied Sciences, Van Yuzuncu Yil University, Türkiye

^2^Department of Pharmaceutical Toxicology, Faculty of Pharmacy, Ege University, İzmir, Türkiye

^3^Department of Pharmaceutical Toxicology, Faculty of Pharmacy, Van Yüzüncü Yıl University, Van, Türkiye

^4^Department of Pharmaceutical Chemistry, Faculty of Pharmacy, Van Yüzüncü Yıl University, Van, Türkiye

^5^Department of Science, Faculty of Educational Sciences, Van Yüzüncü Yil University, Van, Türkiye

*Correspondence: [h_genc2000@yyu.edu.tr](mailto:h_genc2000@yyu.edu.tr)

**Supplementary Material**

| **Contents** | **Pages** |
| --- | --- |
| 1. Structures of the synthesized compounds **13-32** | **2** |
| 1. ^1^H- and ^13^C-NMR spectrum copies of compounds **13-32** | **3** |
| 1. 3D and 2D ligand-protein interactions of PI3K active site with **LASW1579** | **23** |
| 1. 3D and 2D ligand-protein interactions of PI3K active site with compound **21** | **23** |
| 1. 3D and 2D ligand-protein interactions of PI3K active site with compound **26** | **24** |
| 1. 3D and 2D ligand-protein interactions of PI3K active site with compound **27** | **24** |
| 1. HRMS spectrum copies of compounds **13-32** | **25** |

- - - 1. Structures of the synthesized compounds **13-32**

- - - 1. ^1^H- and ^13^C-NMR spectrum copies of compounds **13-12**


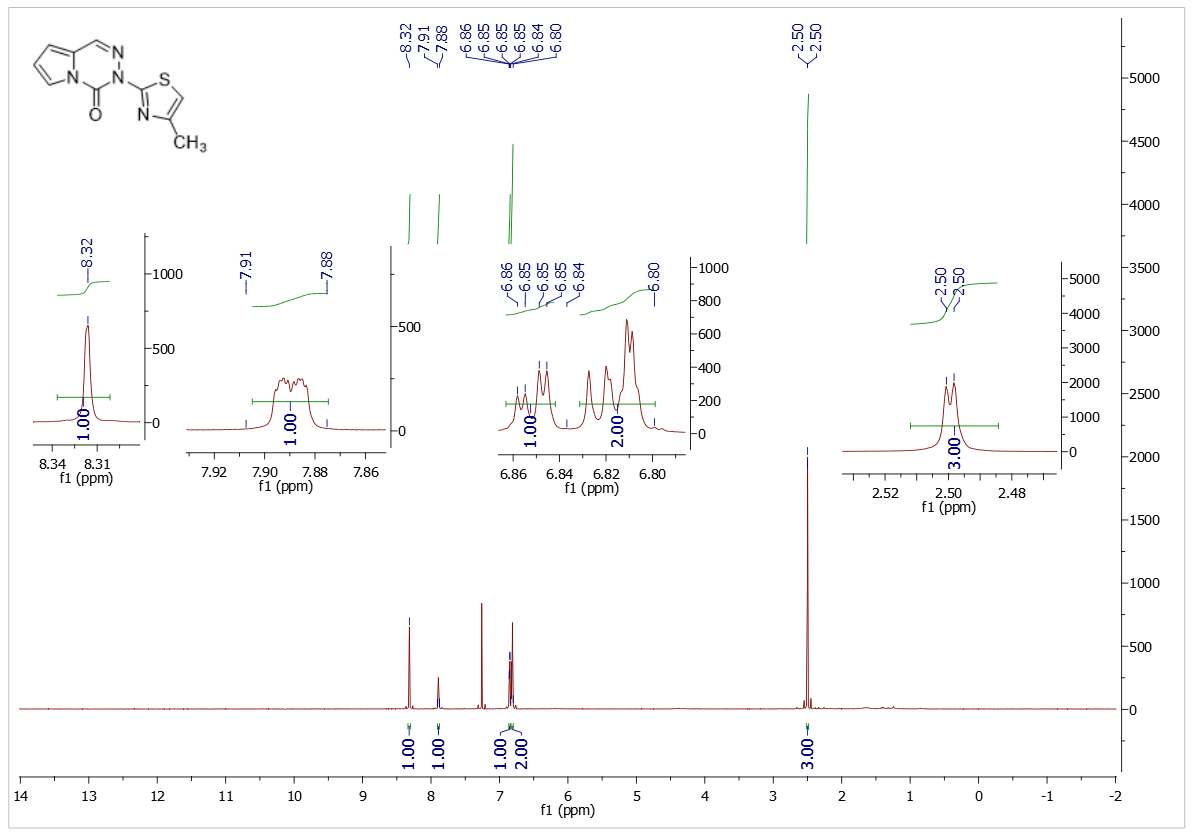


**Figure S1.** ^1^H NMR spectrum of compound **13**


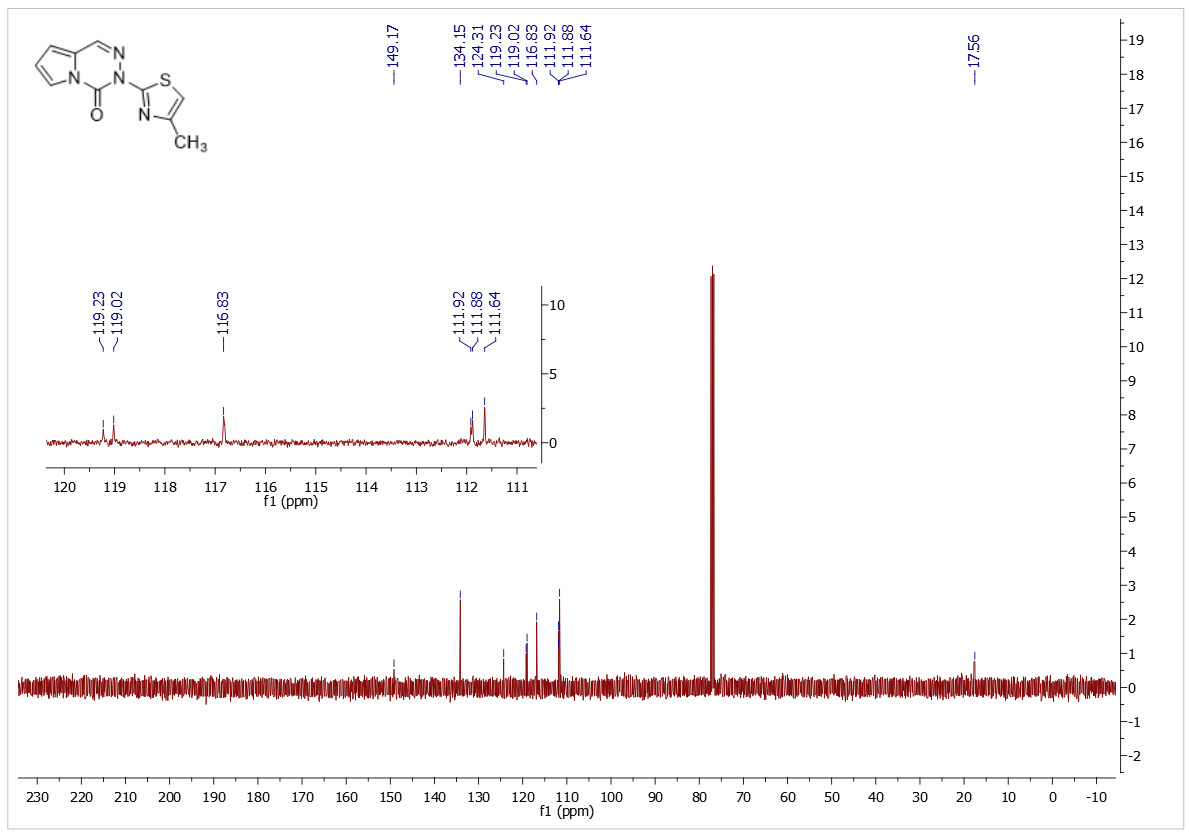


**Figure S2.** ^13^C NMR spectrum of compound **13**


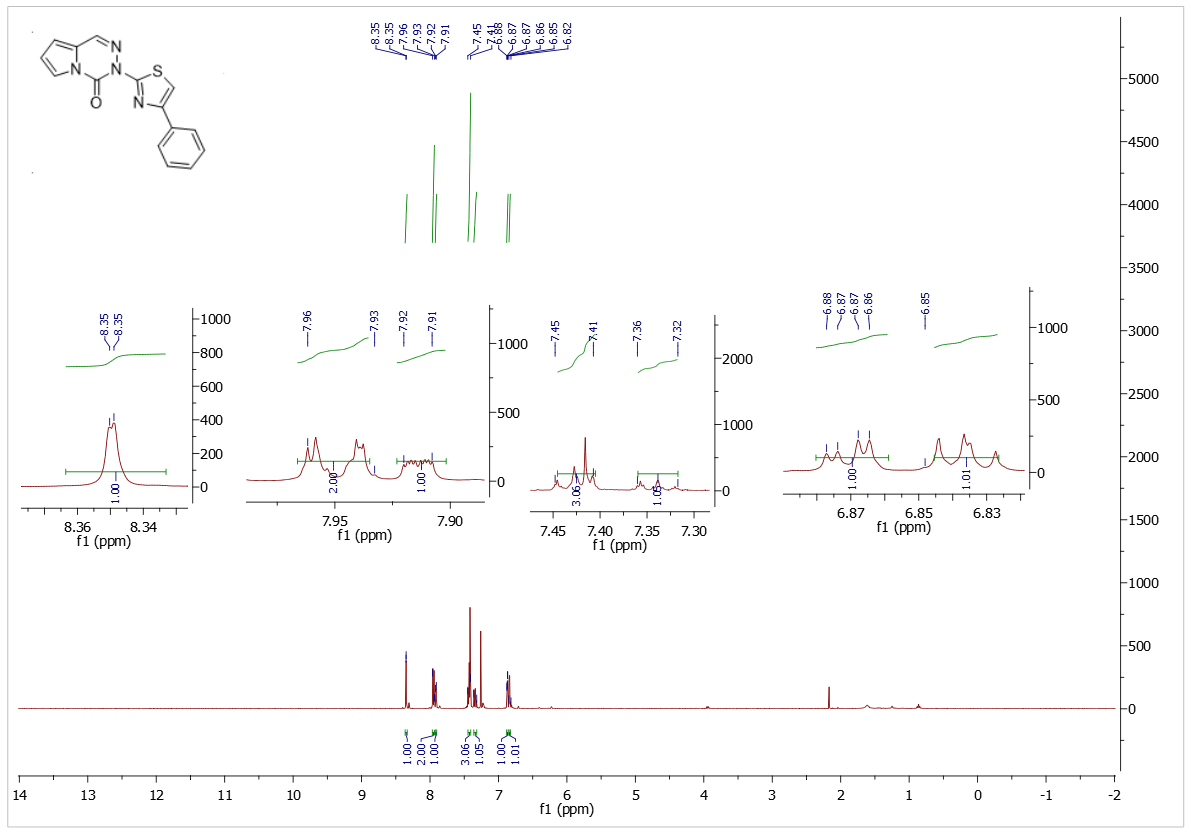


**Figure S3.** ^1^H NMR spectrum of compound **14**


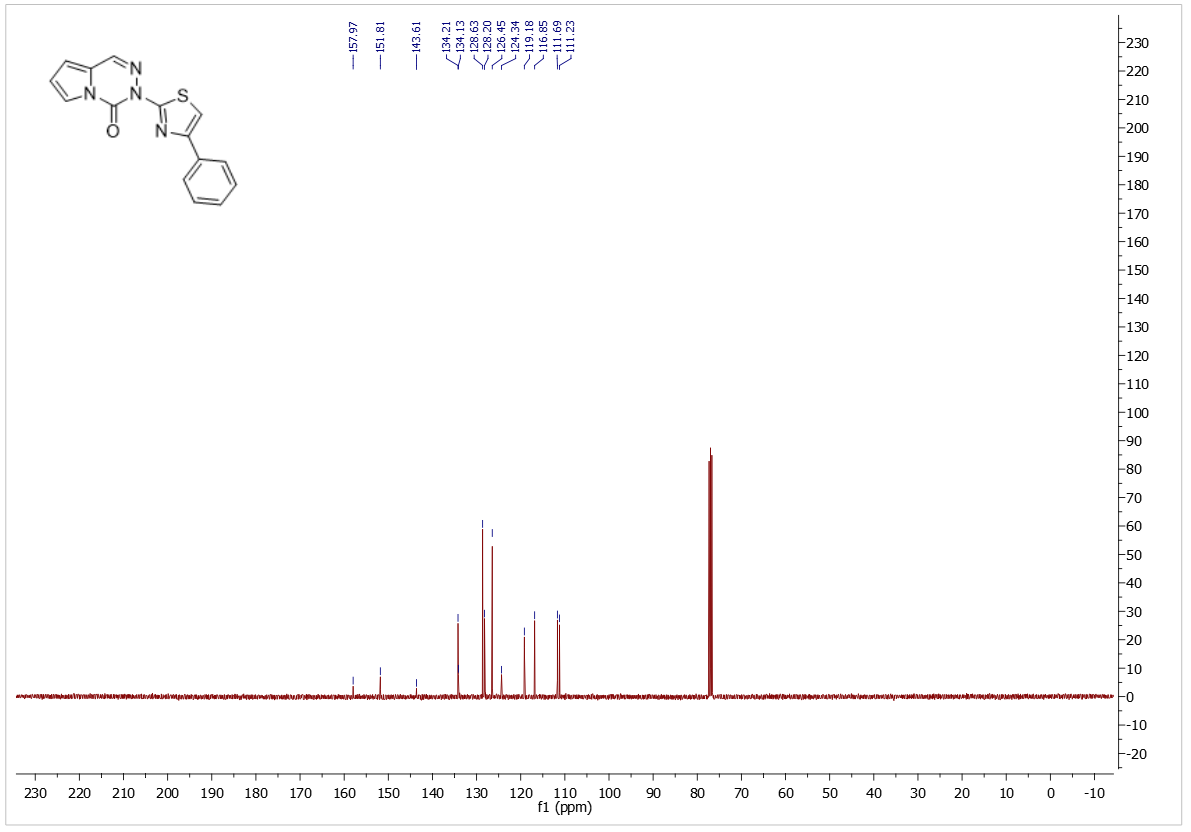


**Figure S4.** ^13^C NMR spectrum of compound **14**


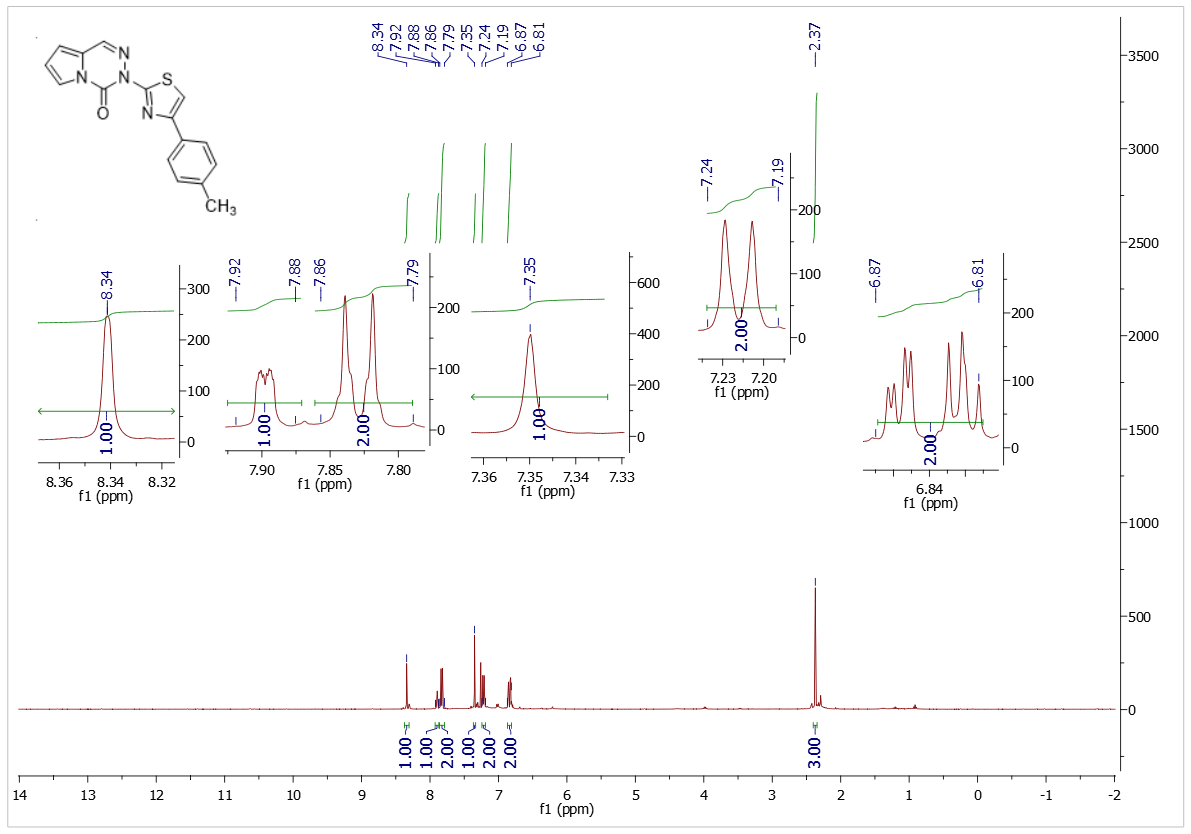


**Figure S5.** ^1^H NMR spectrum of compound **15**
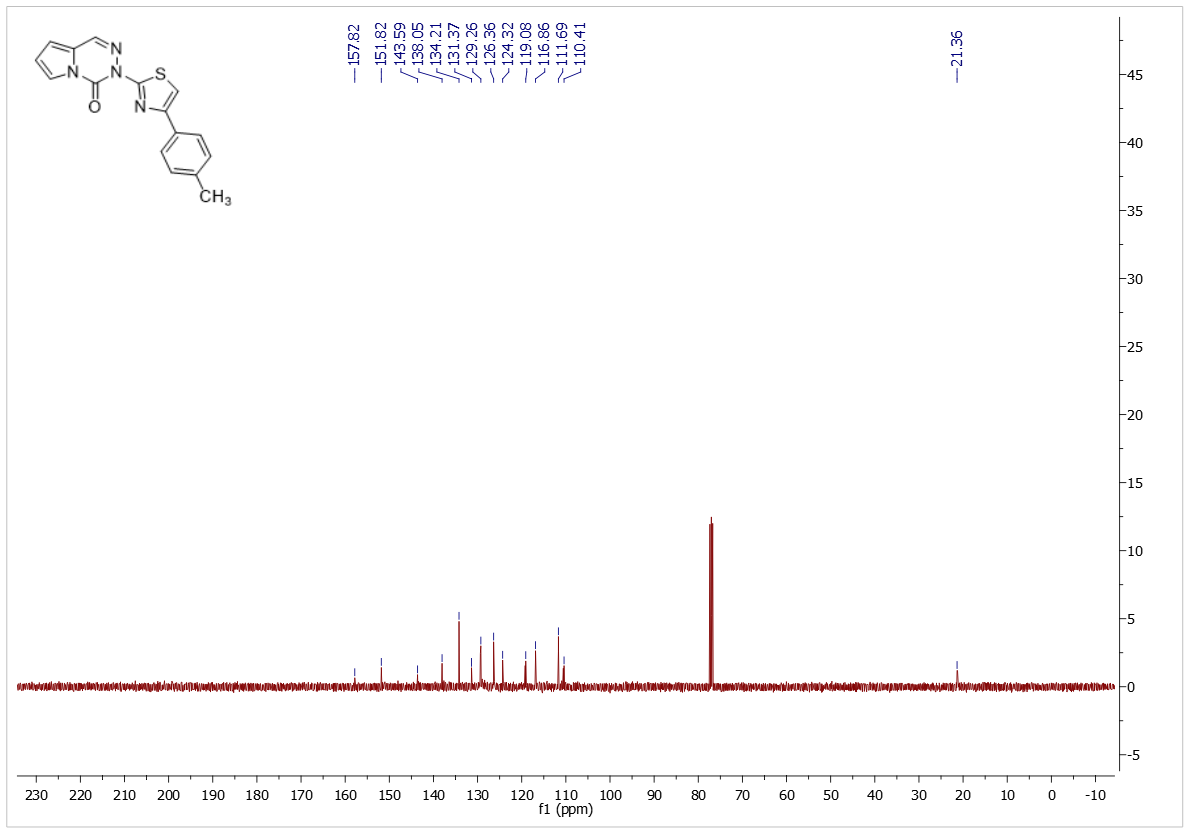


**Figure S6.** ^13^C NMR spectrum of compound **15**


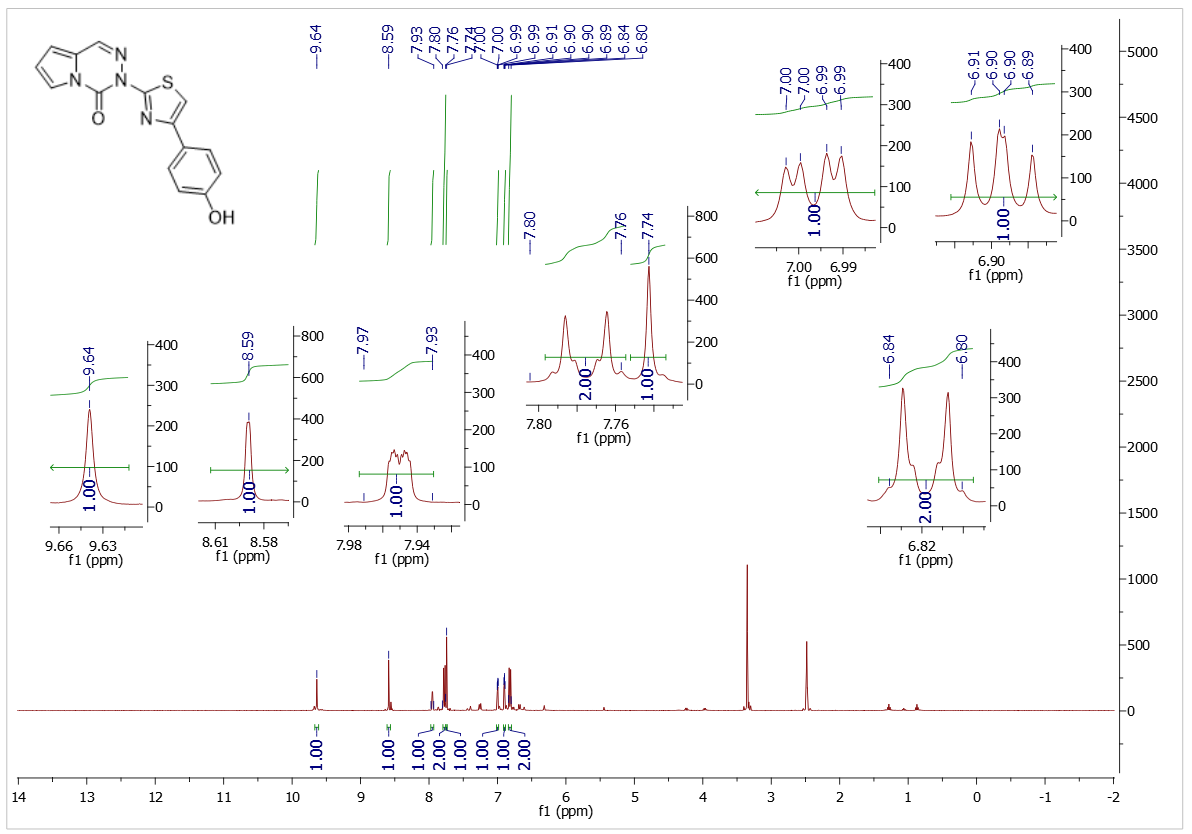


**Figure S7** ^1^H NMR spectrum of compound **16**


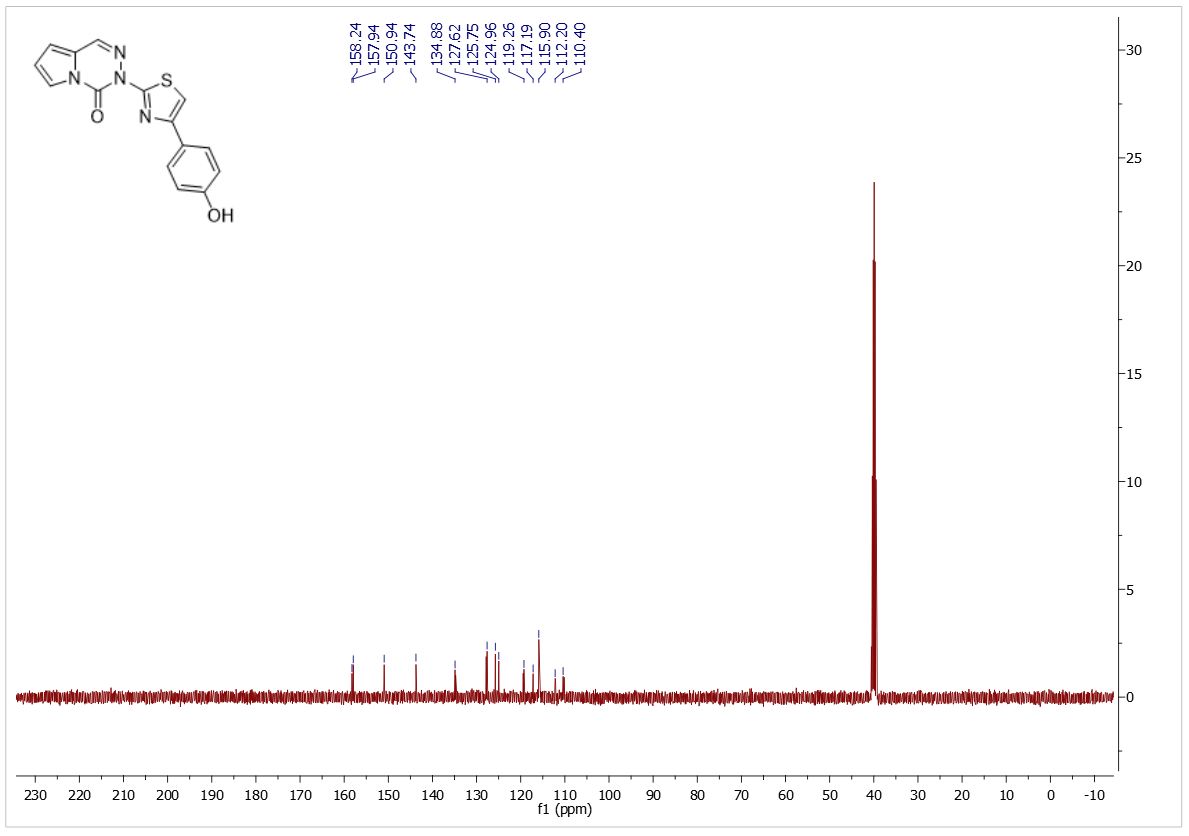


**Figure S8.** ^13^C NMR spectrum of compound **16**


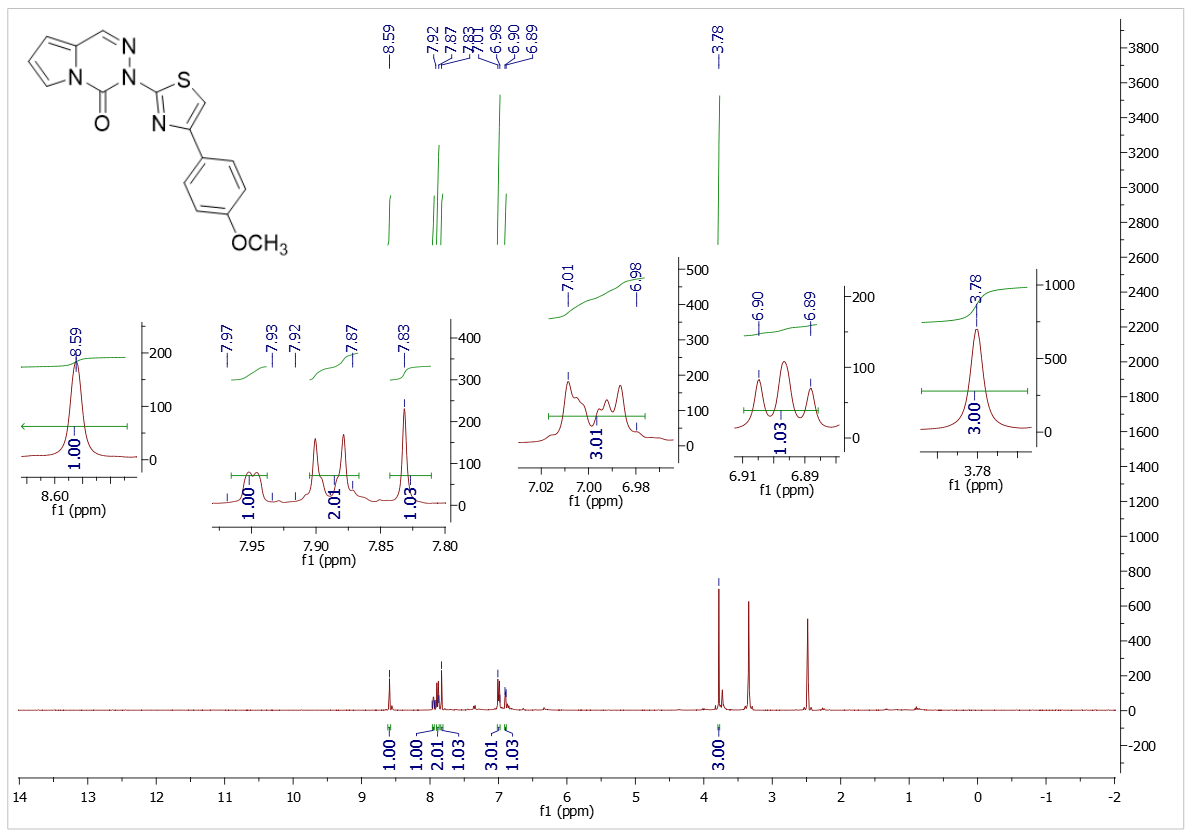


**Figure S9.** ^1^H NMR spectrum of compound **17**


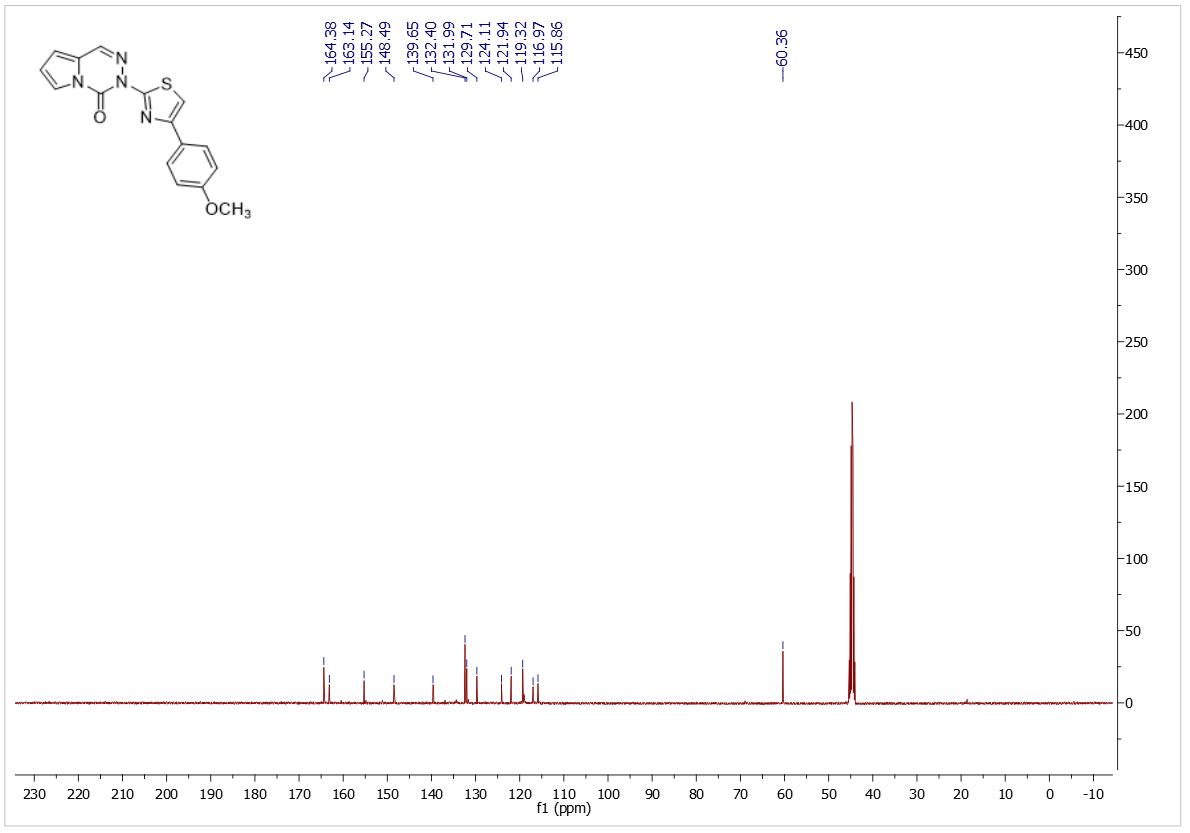


**Figure S10.** ^13^C NMR spectrum of compound **17**


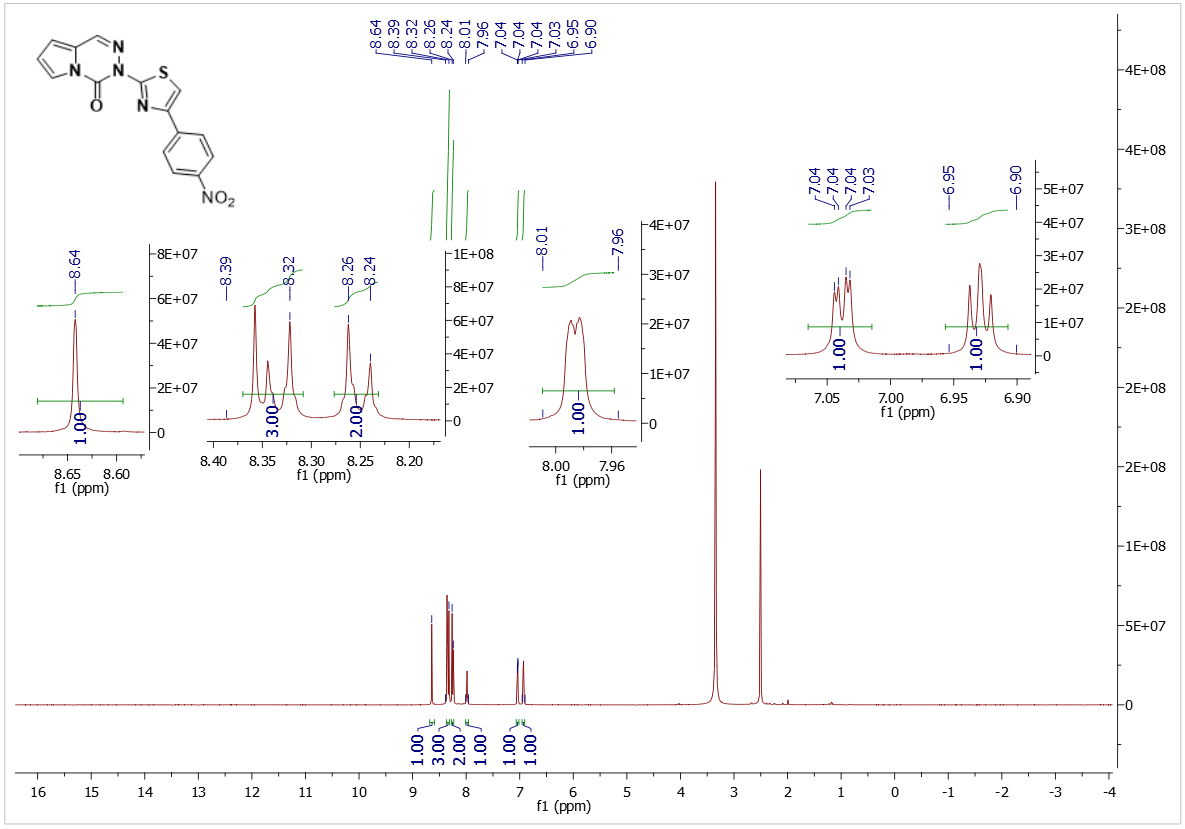


**Figure S11.** ^1^H NMR spectrum of compound **18**


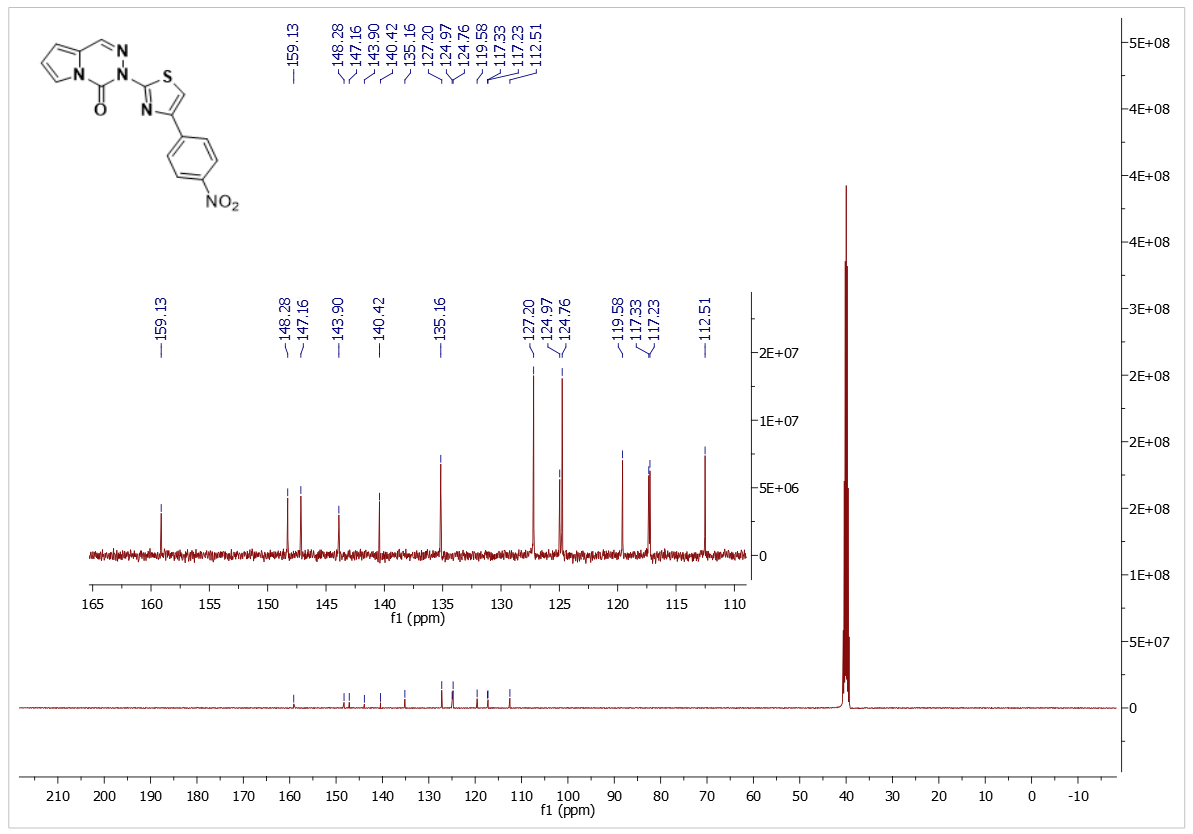


**Figure S12.** ^13^C NMR spectrum of compound **18**


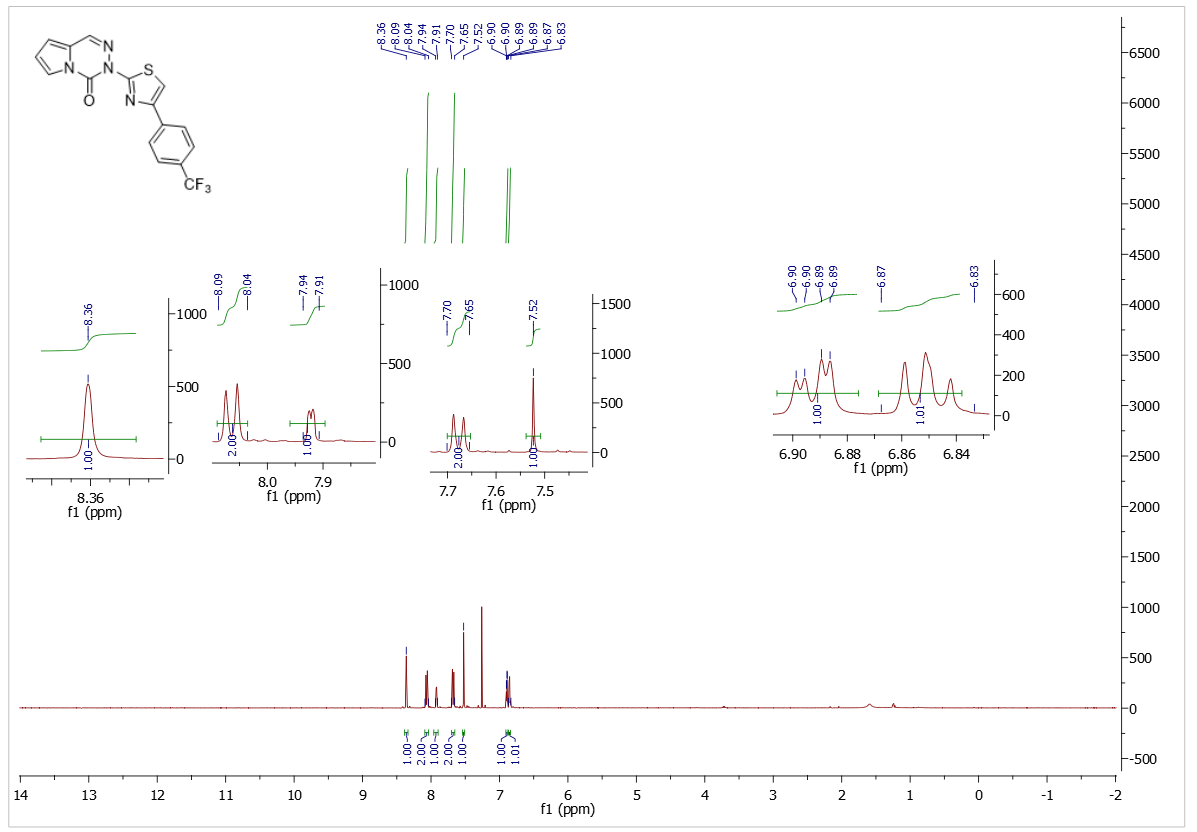


**Figure S13.** ^1^H NMR spectrum of compound **19**


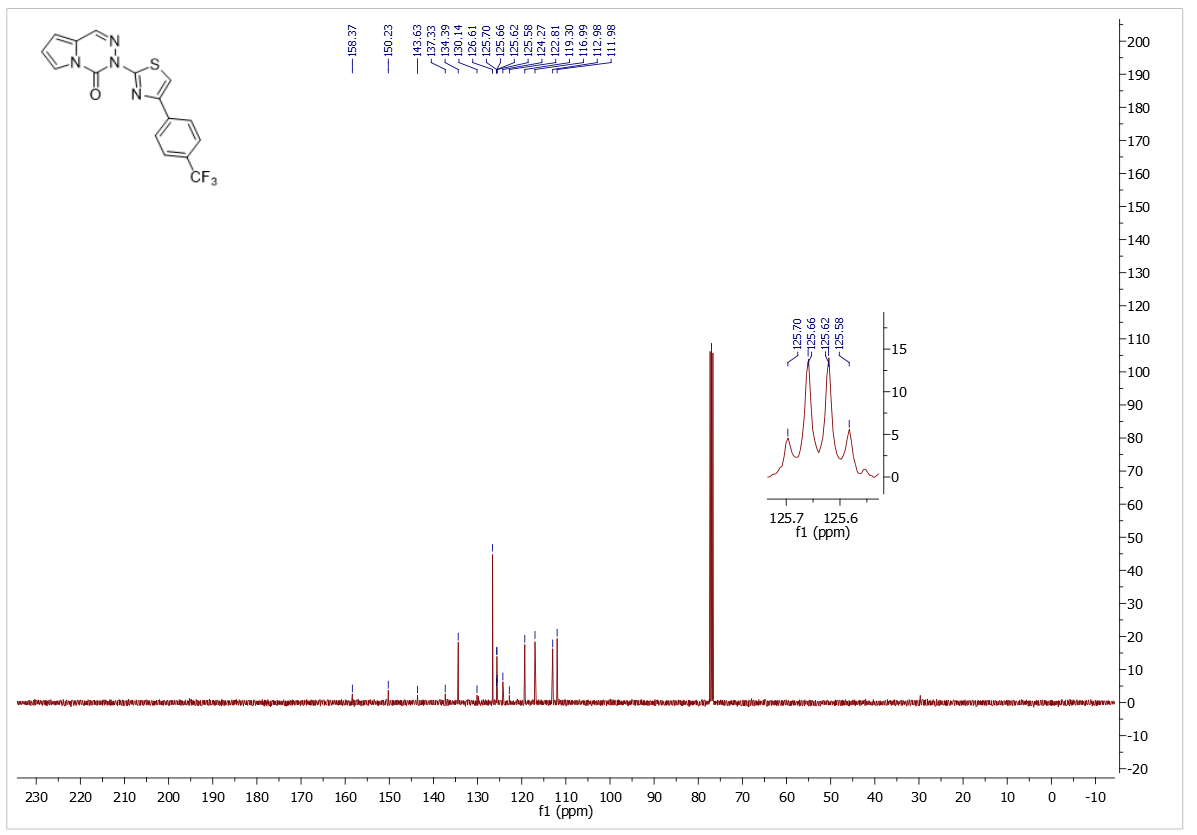


**Figure S14.** ^13^C NMR spectrum of compound **19**


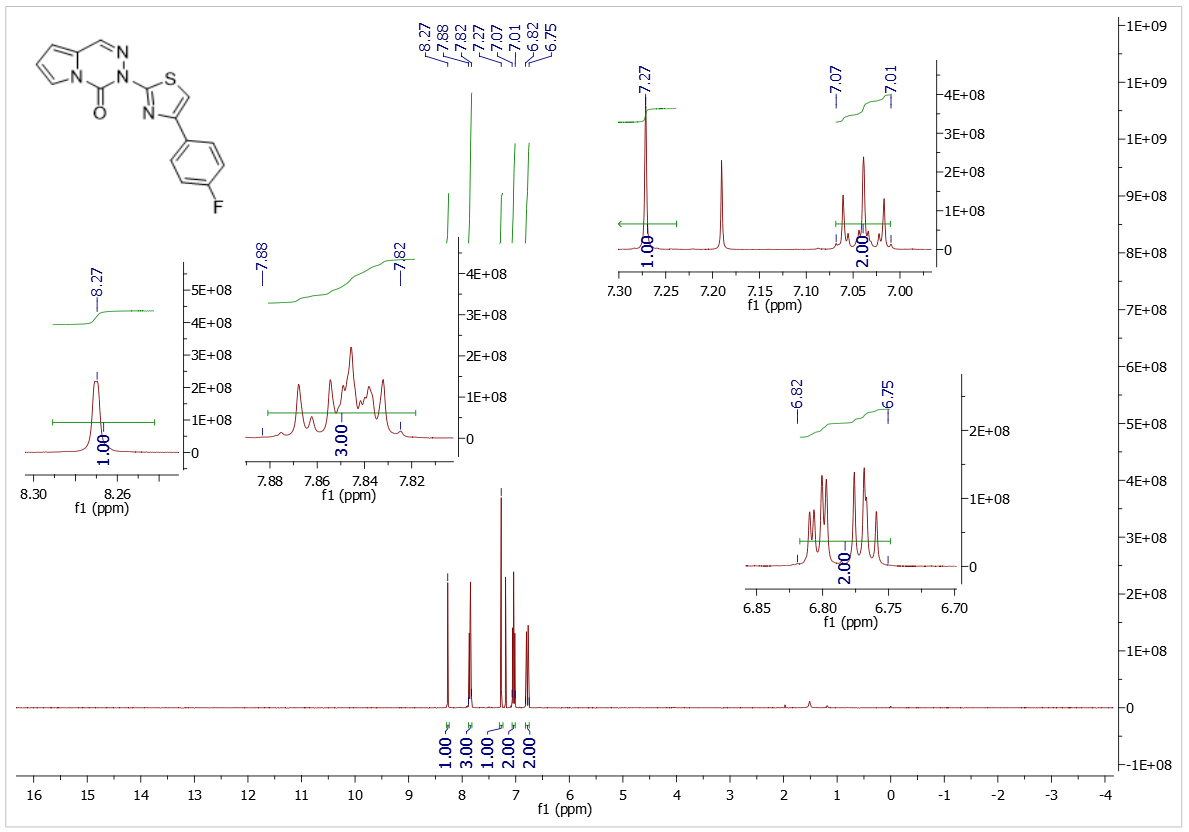


**Figure S15.** ^1^H NMR spectrum of compound **20**


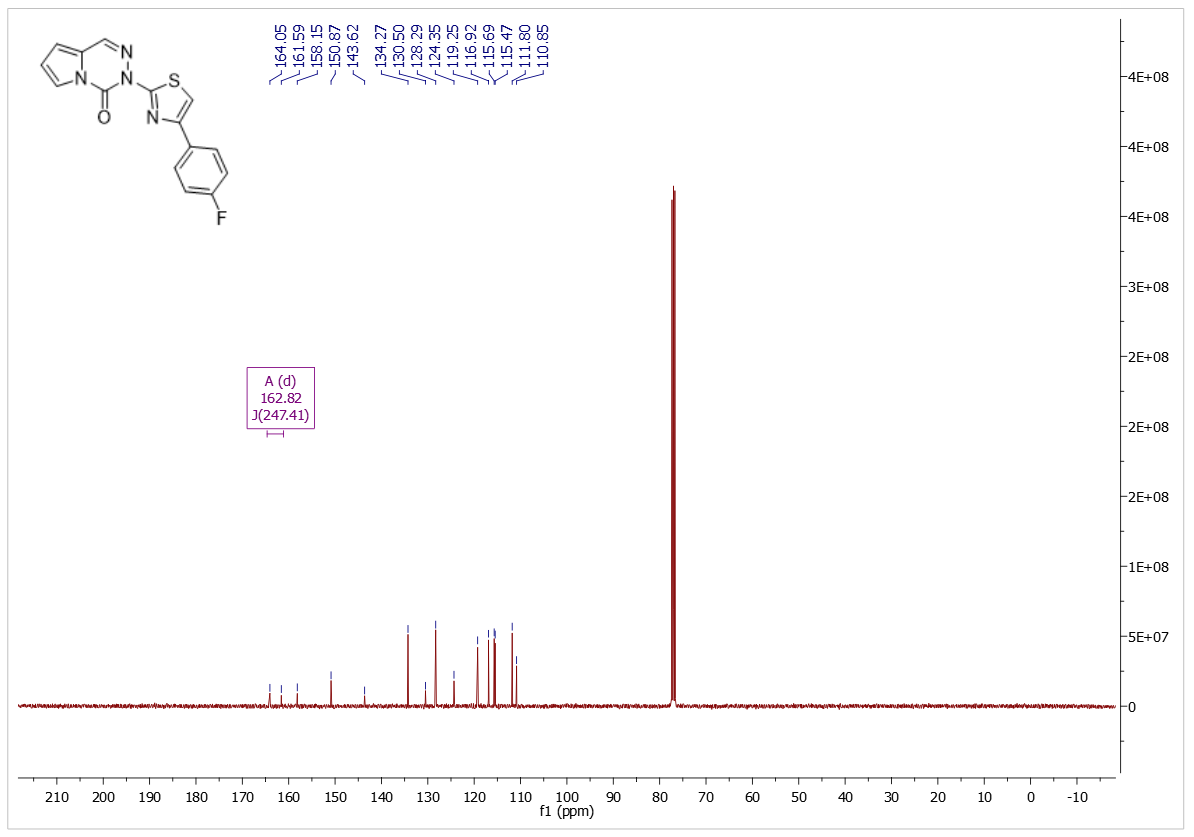


**Figure S16.** ^13^C NMR spectrum of compound **20**


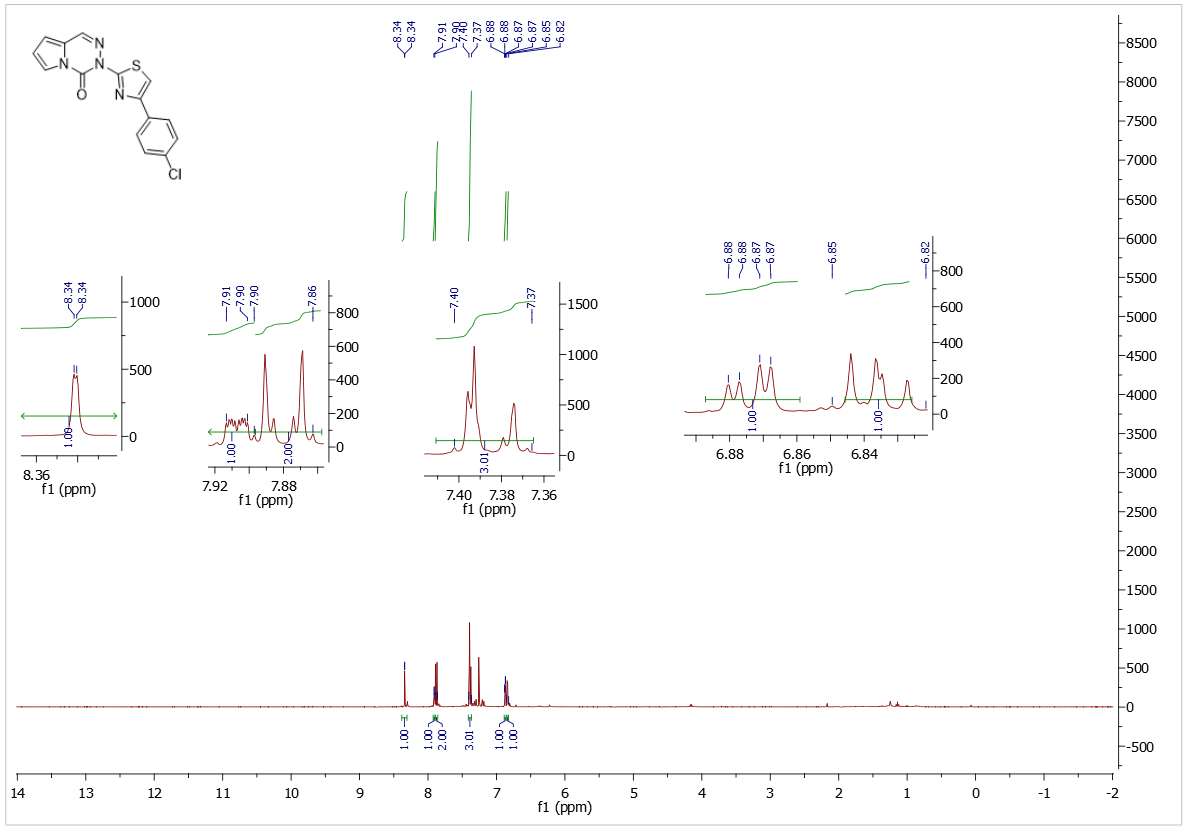


**Figure S17.** ^1^H NMR spectrum of compound **21**


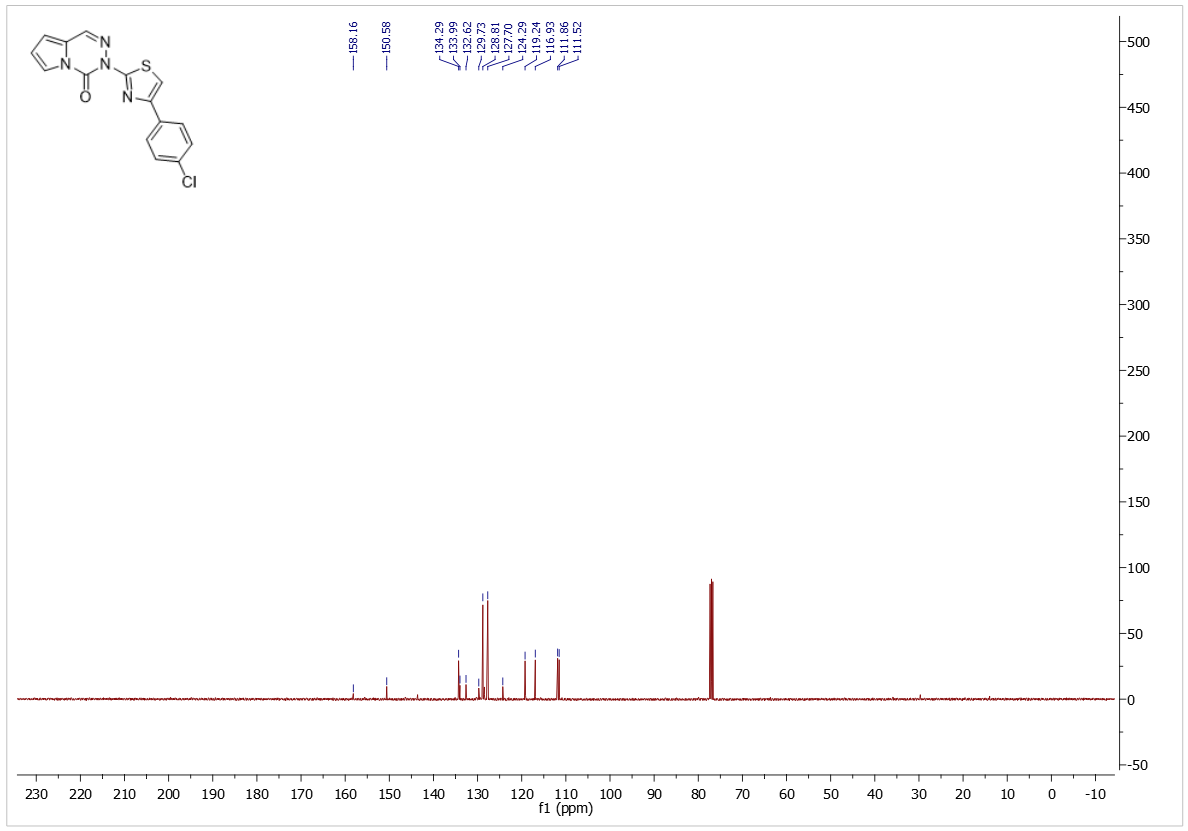


**Figure S18.** ^13^C NMR spectrum of compound **21**


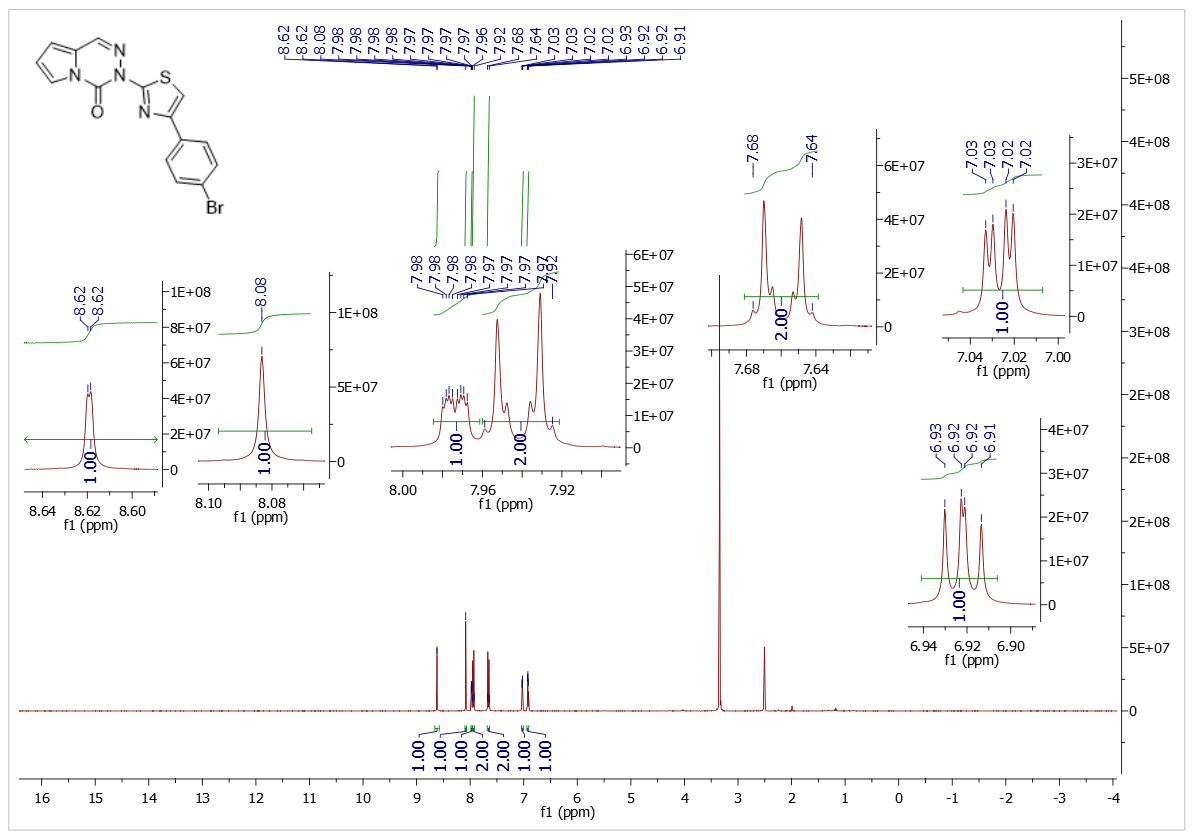


**Figure S19.** ^1^H NMR spectrum of compound **22**


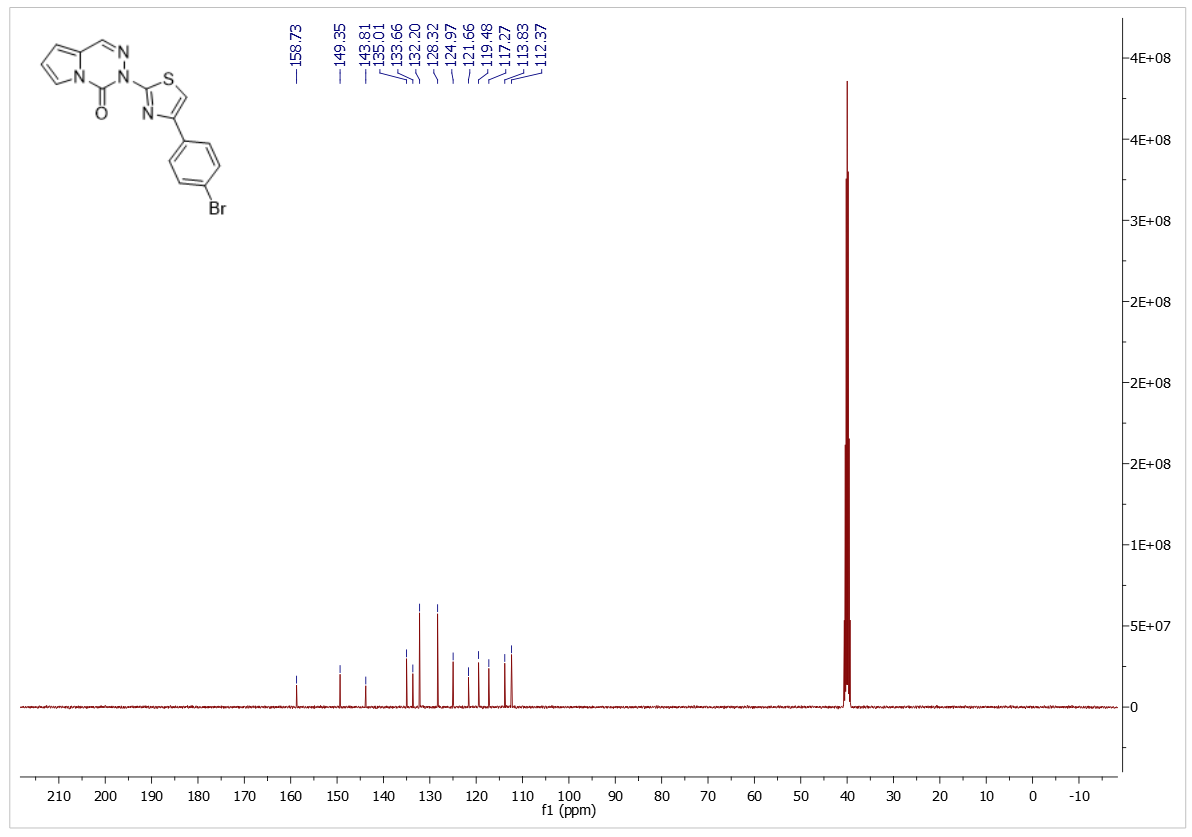


**Figure S20.** ^13^C NMR spectrum of compound **22**


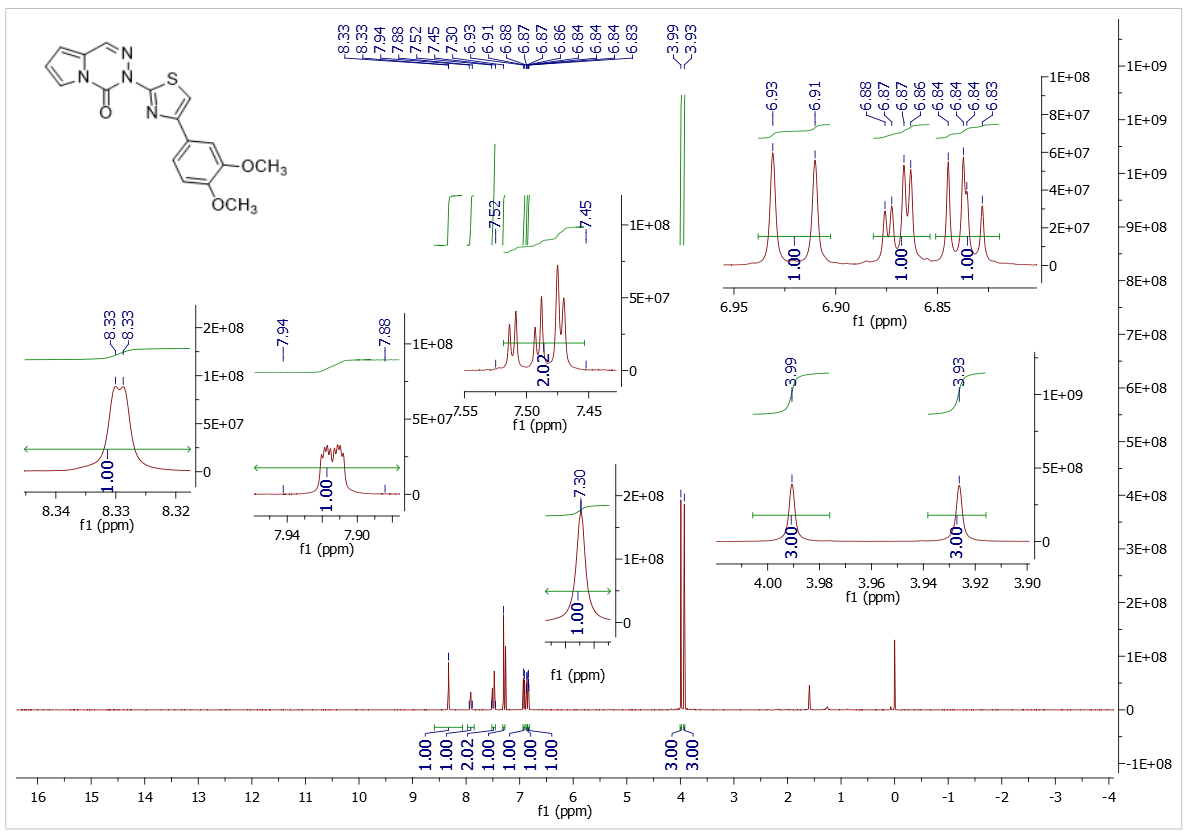


**Figure S21.** ^1^H NMR spectrum of compound **23**


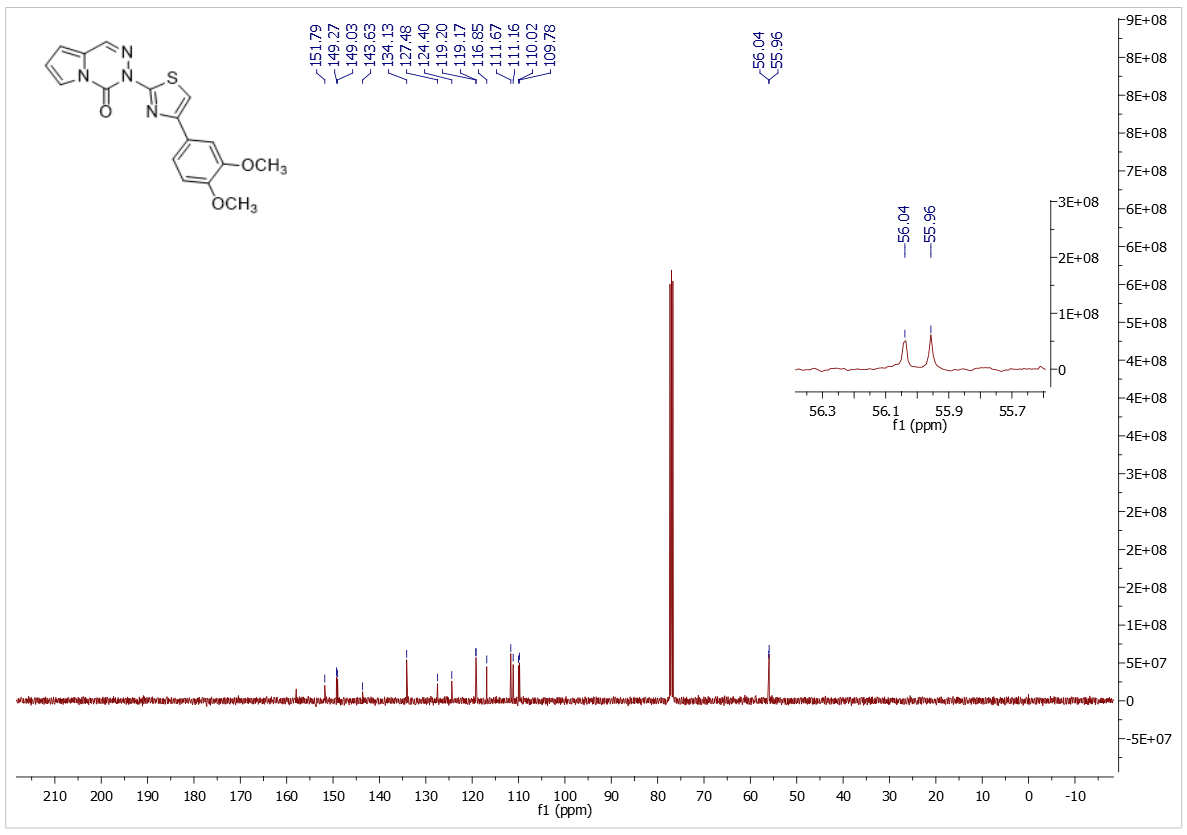


**Figure S22.** ^13^C NMR spectrum of compound **23**


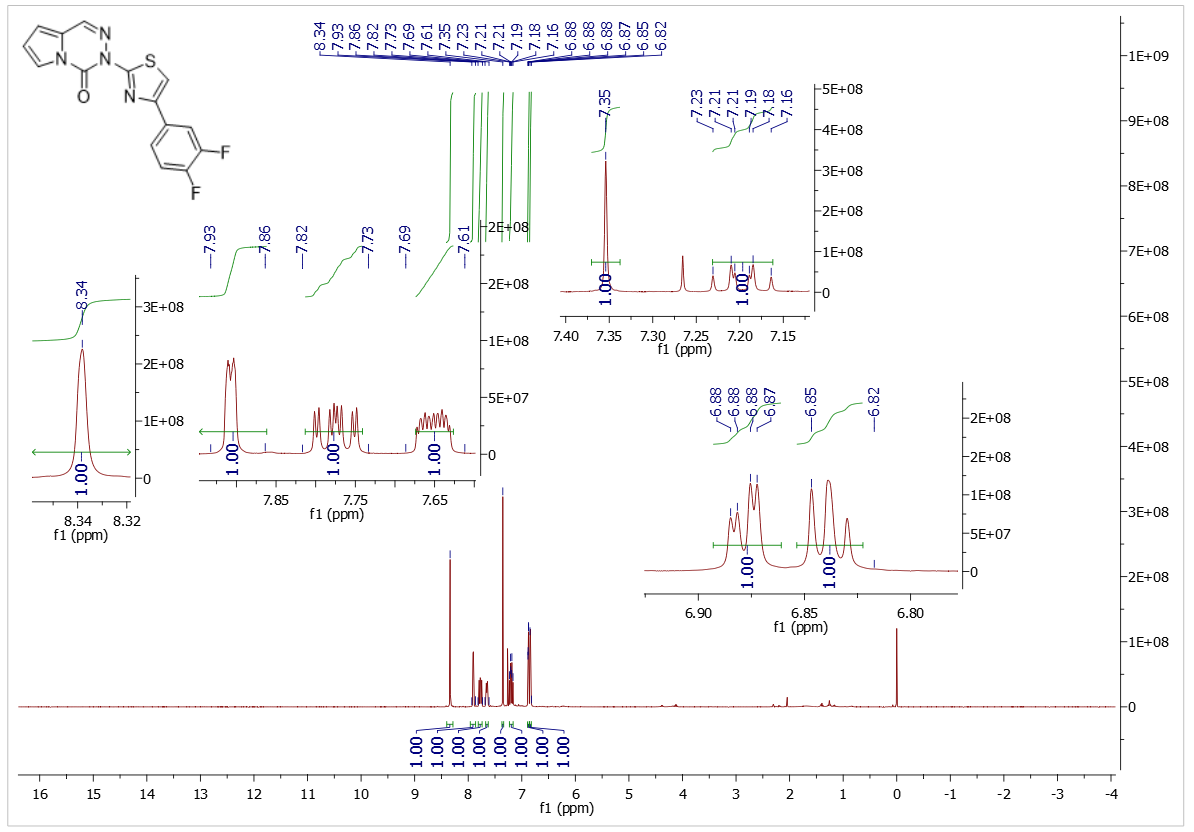


**Figure S23.** ^1^H NMR spectrum of compound **24**


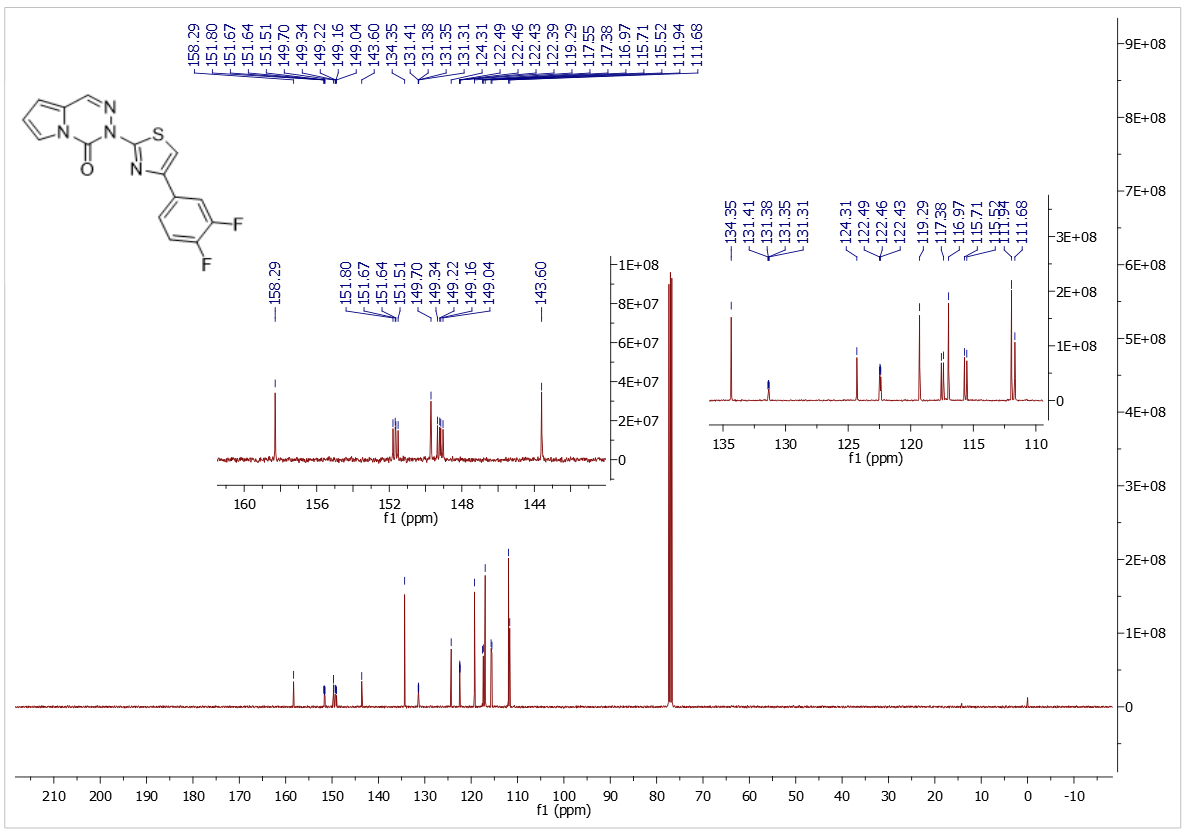


**Figure S24.** ^13^C NMR spectrum of compound **24**


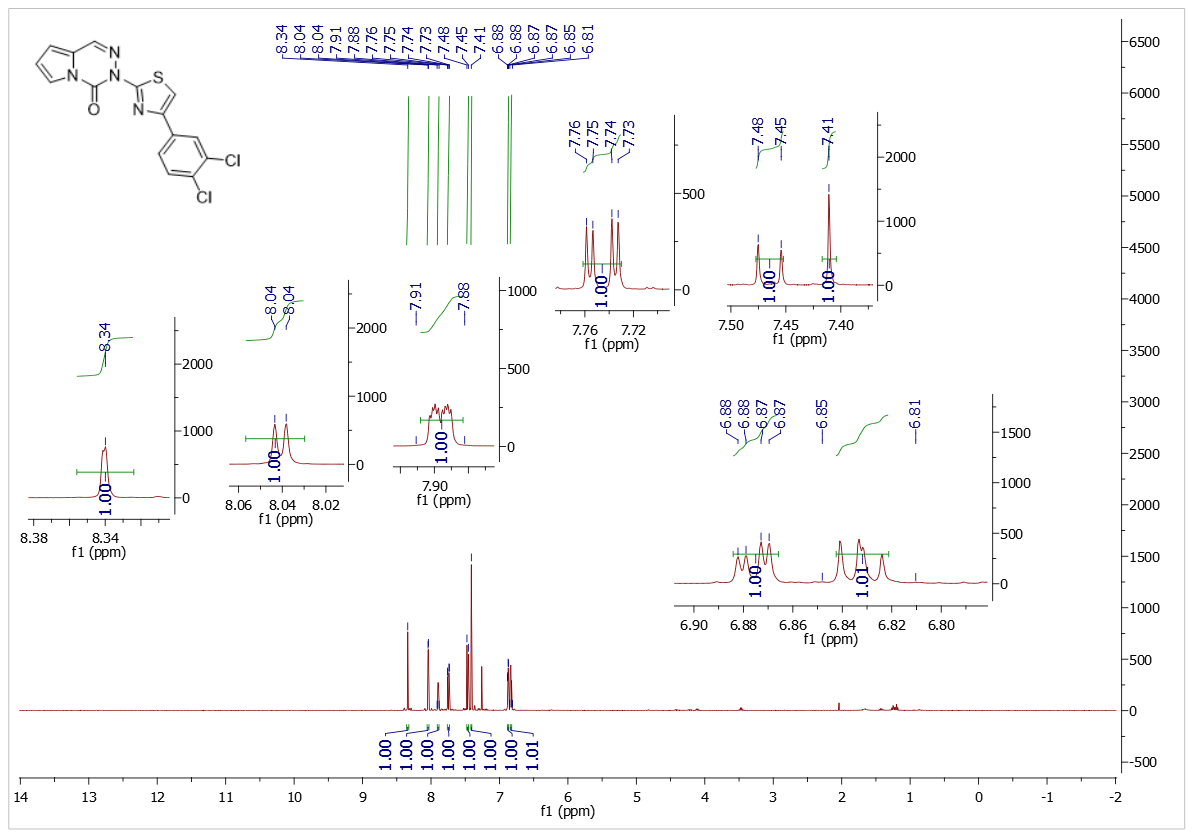


**Figure S25.** ^1^H NMR spectrum of compound **25**


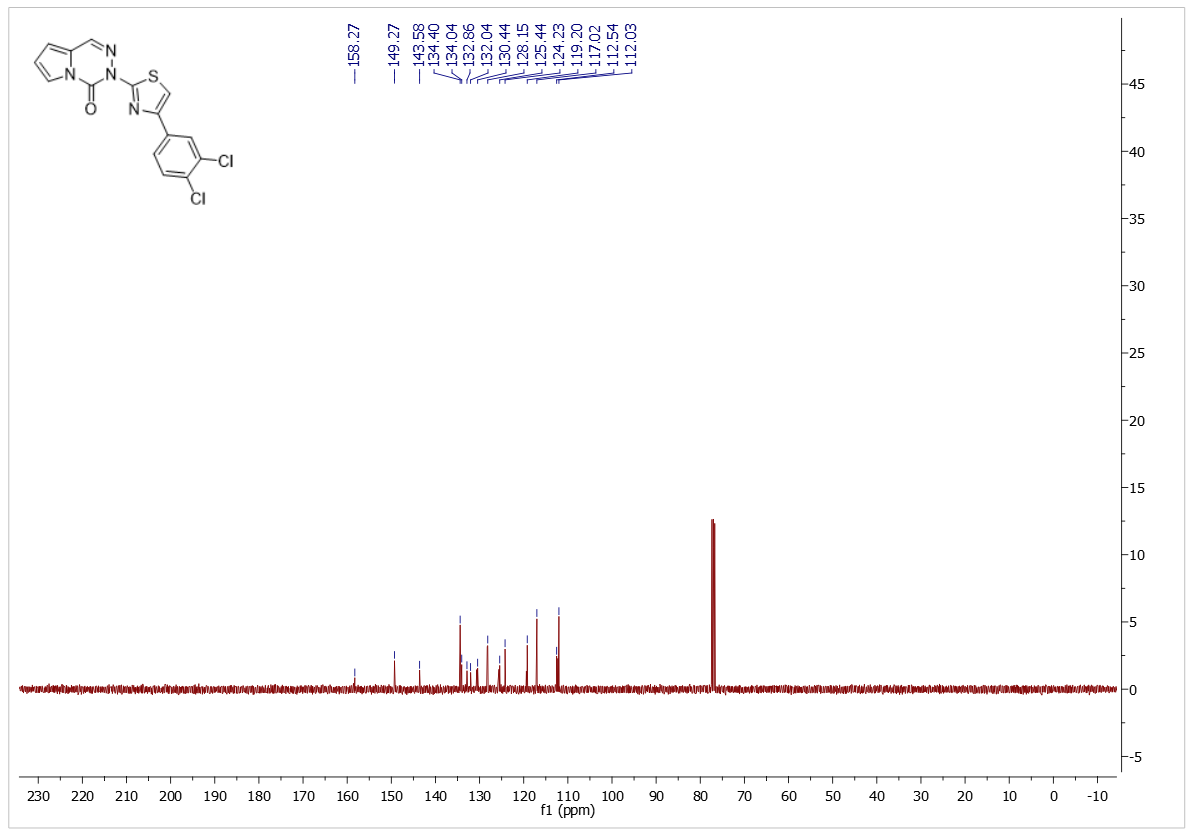


**Figure S26.** ^13^C NMR spectrum of compound **25**


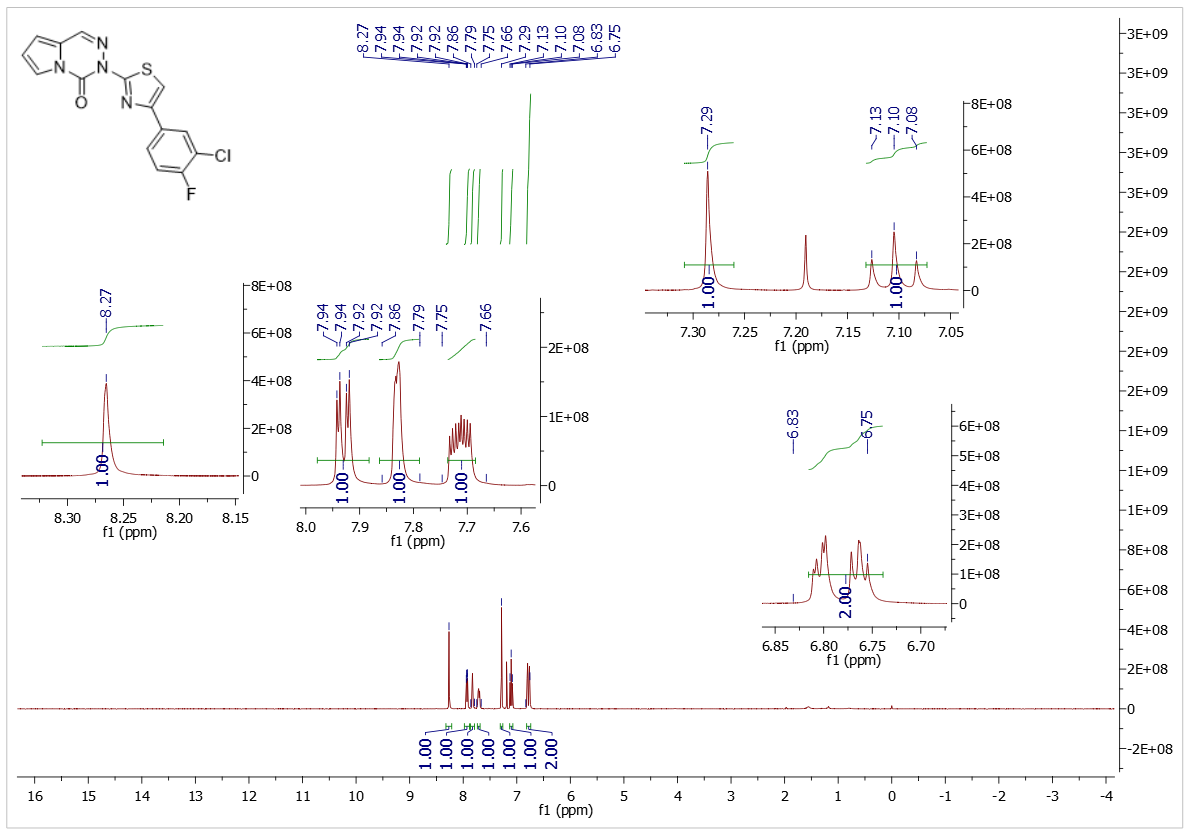


**Figure S27.** ^1^H NMR spectrum of compound **26**


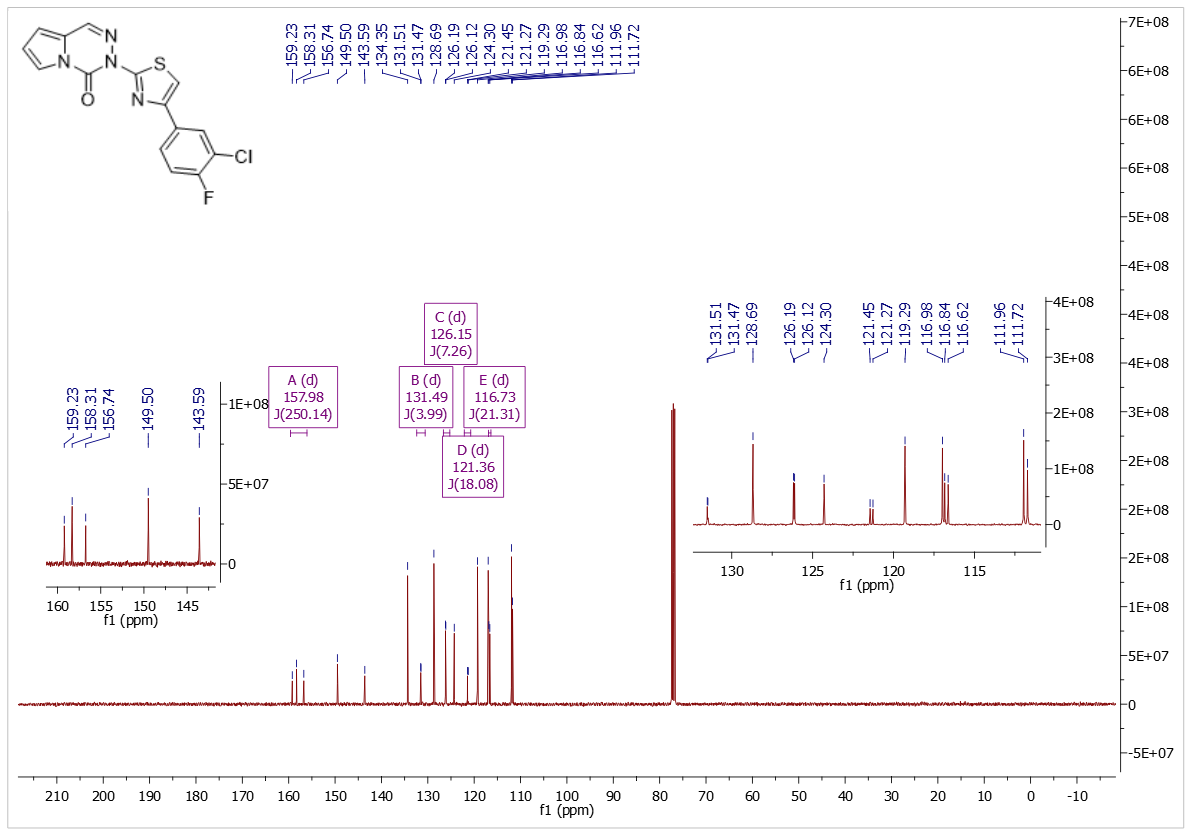


**Figure S28.** ^13^C NMR spectrum of compound **26**


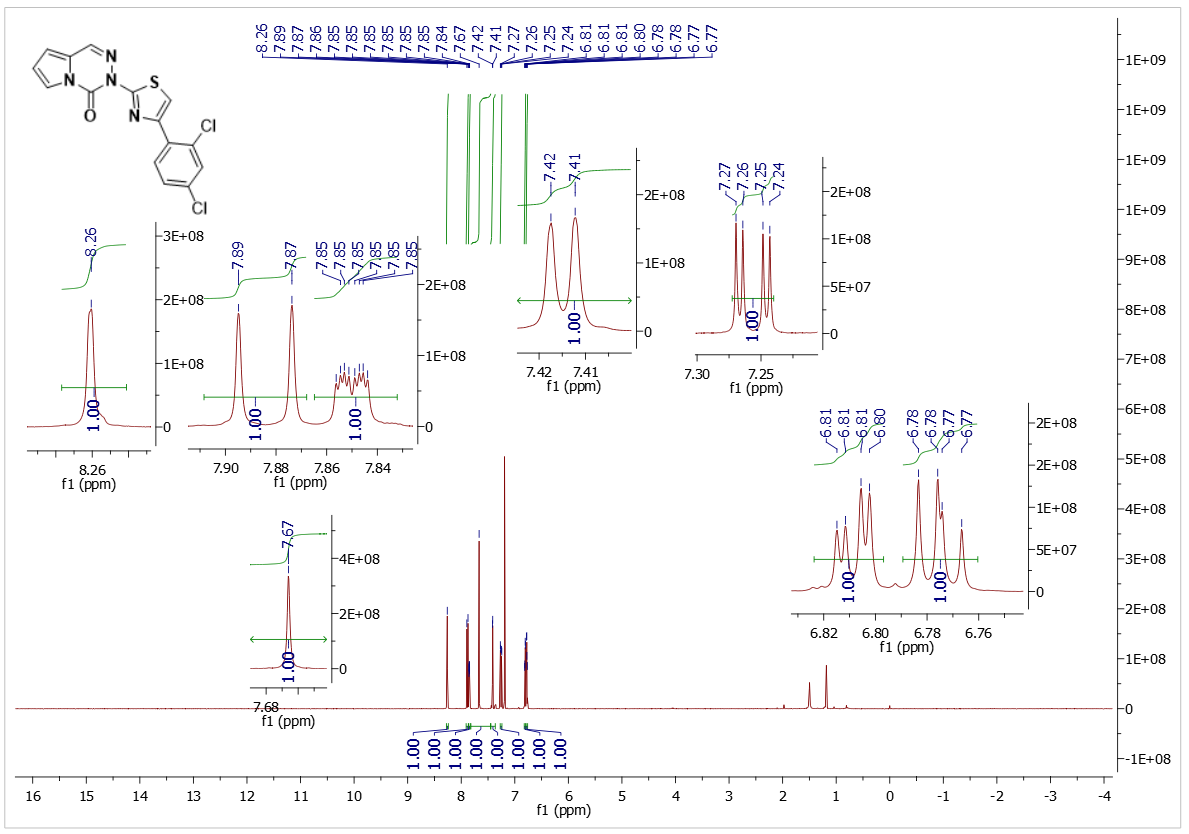


**Figure S29.** ^1^H NMR spectrum of compound **27**


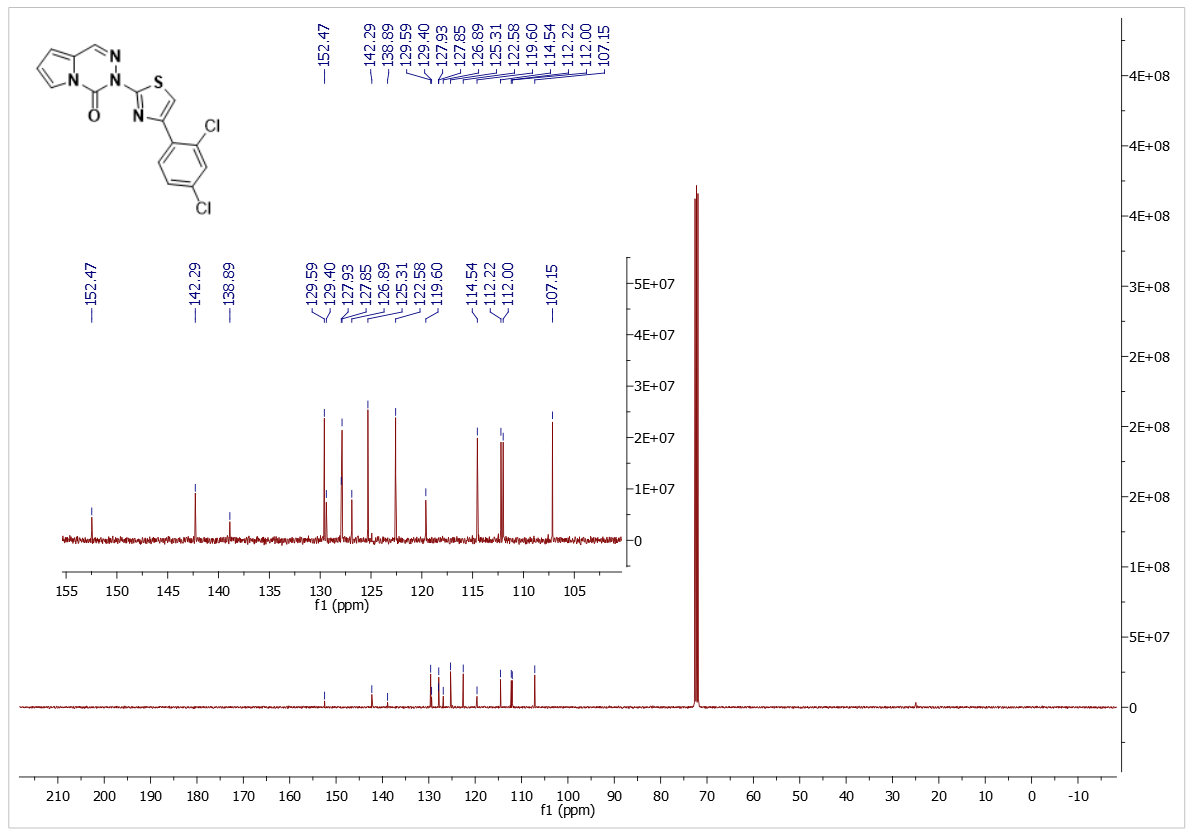


**Figure S30.** ^13^C NMR spectrum of compound **27**


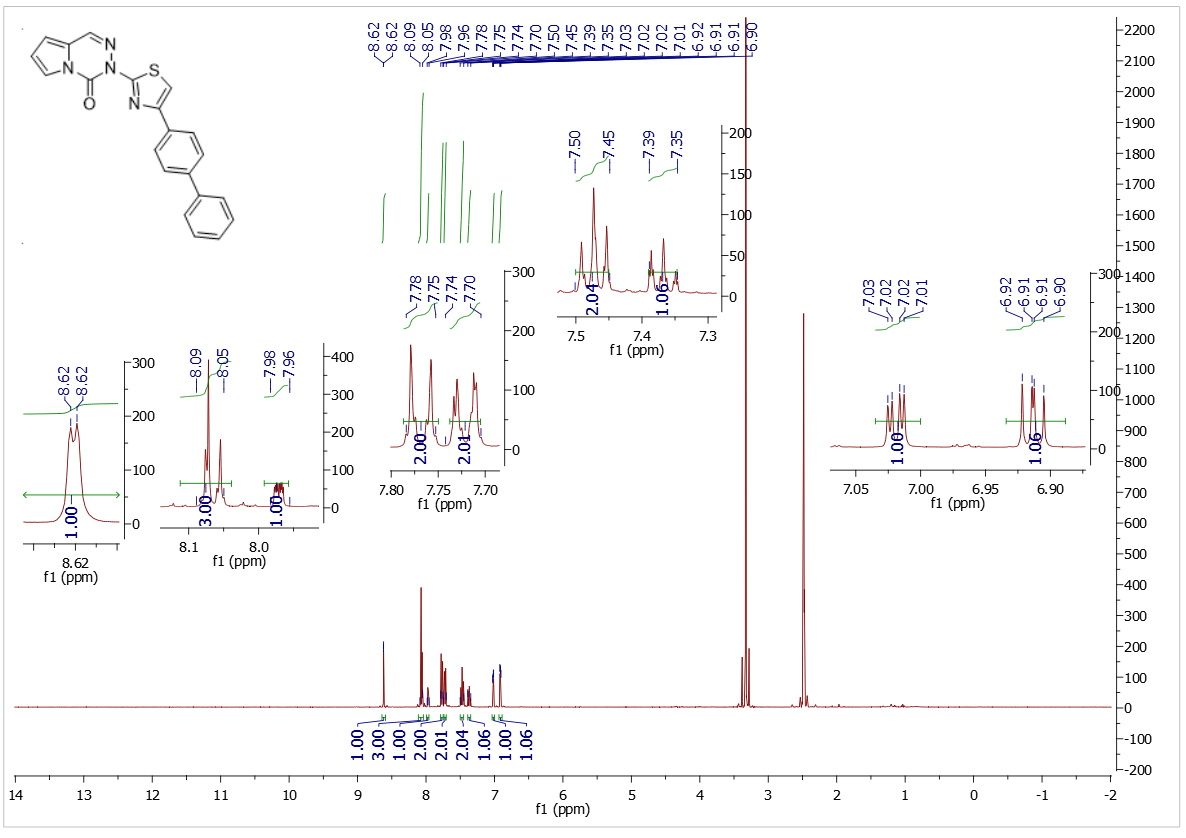


**Figure S31.** ^1^H NMR spectrum of compound **28**


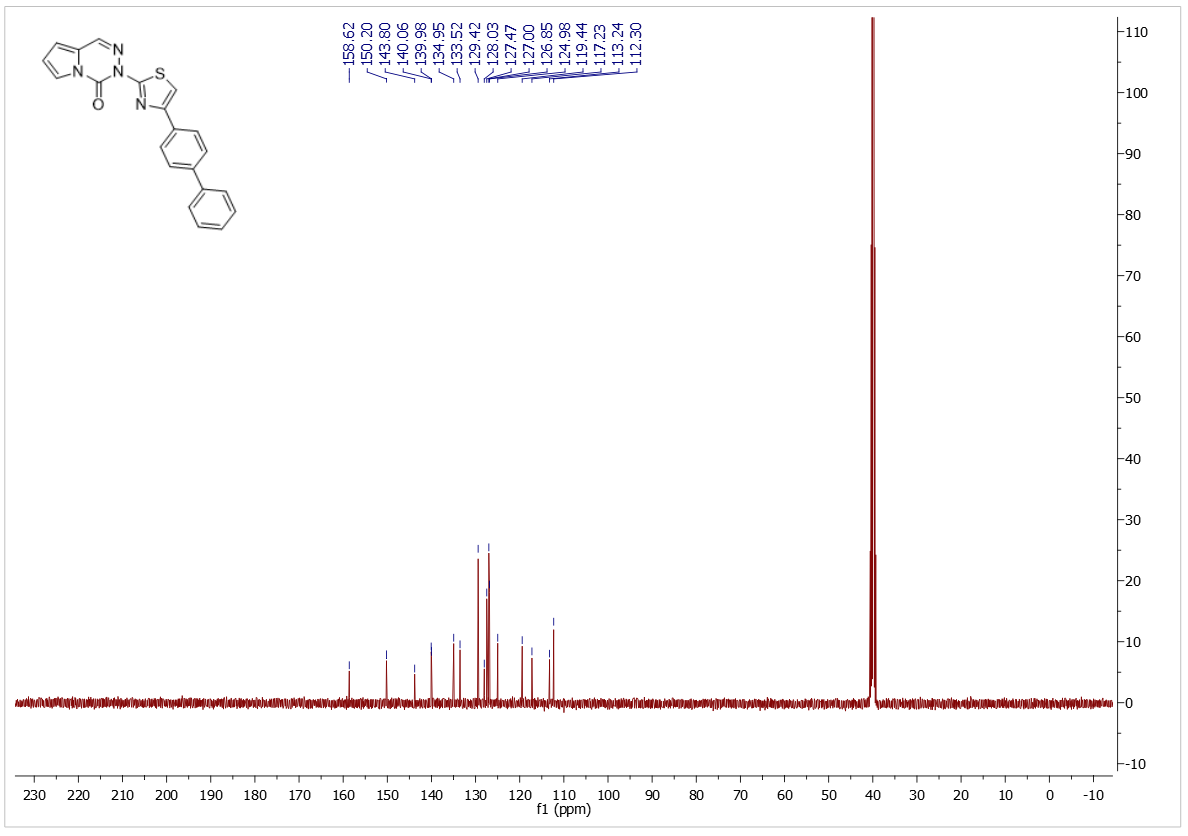


**Figure S32.** ^13^C NMR spectrum of compound **28**


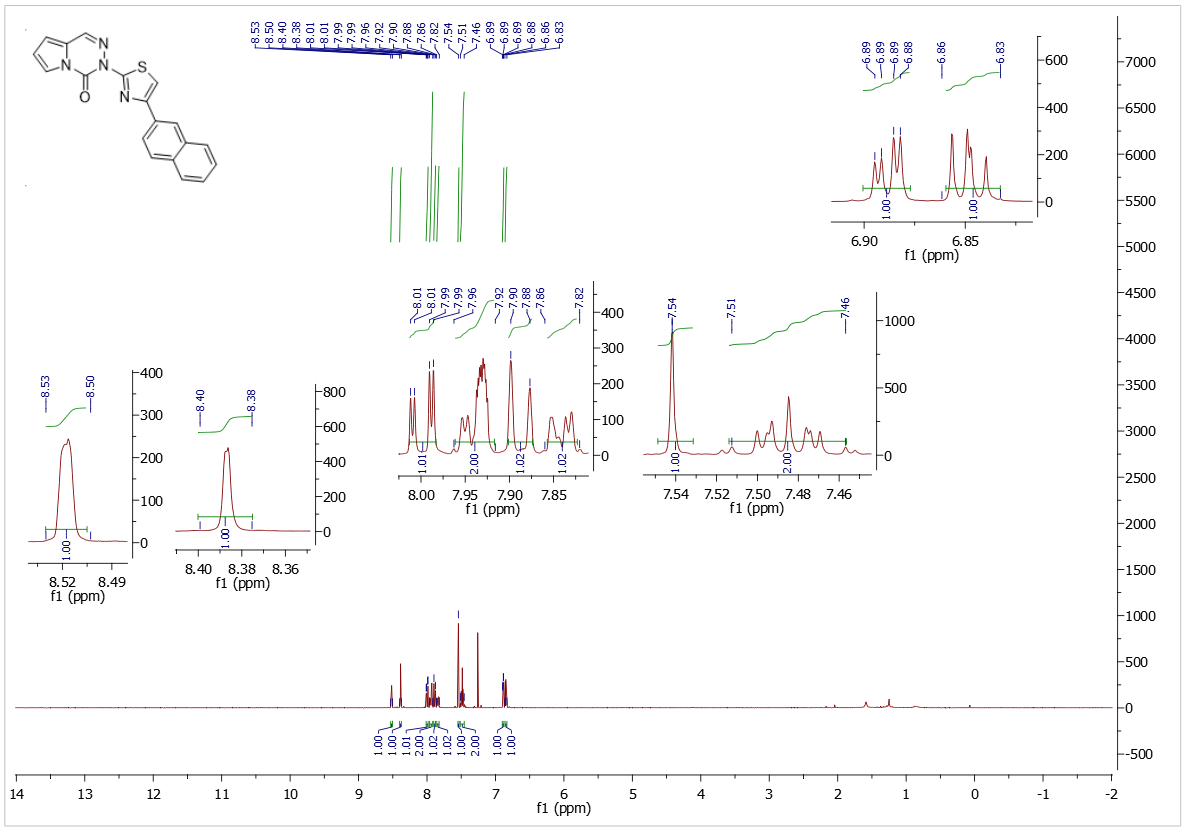


**Figure S33.** ^1^H NMR spectrum of compound **29**


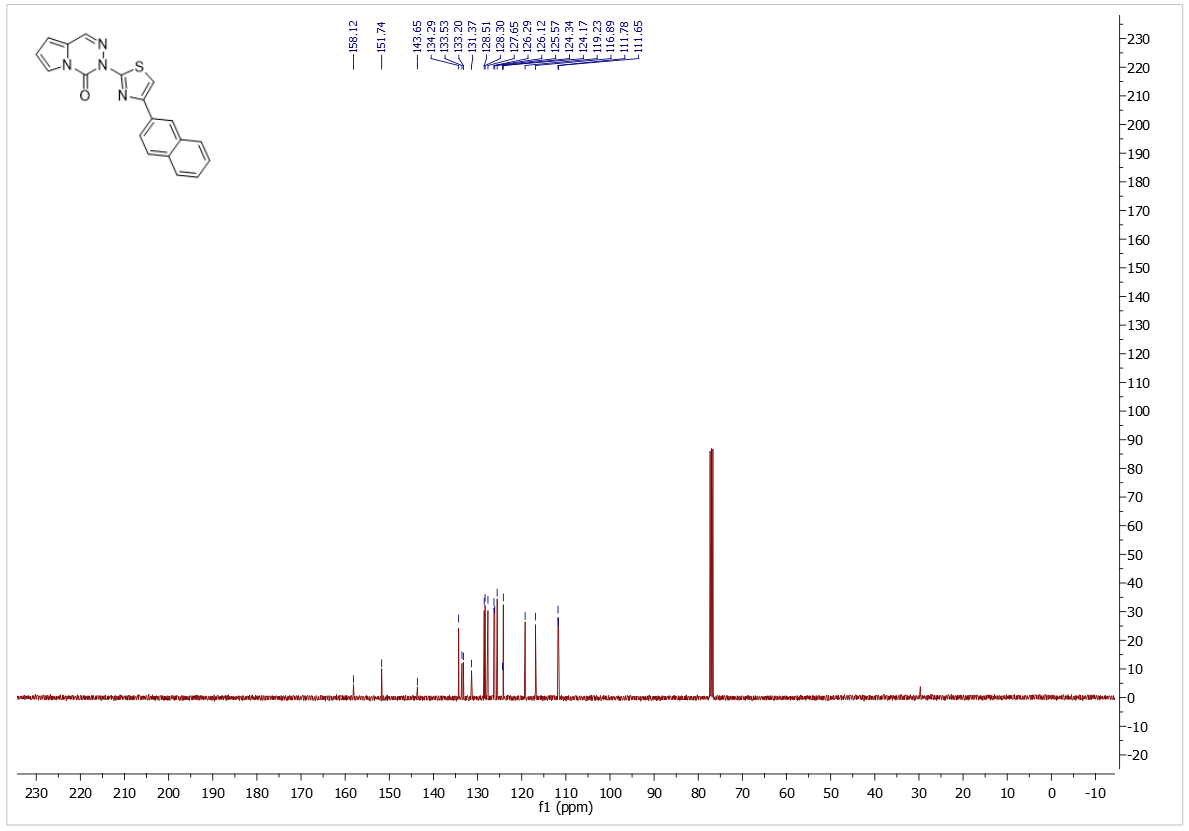


**Figure S34.** ^13^C NMR spectrum of compound **29**


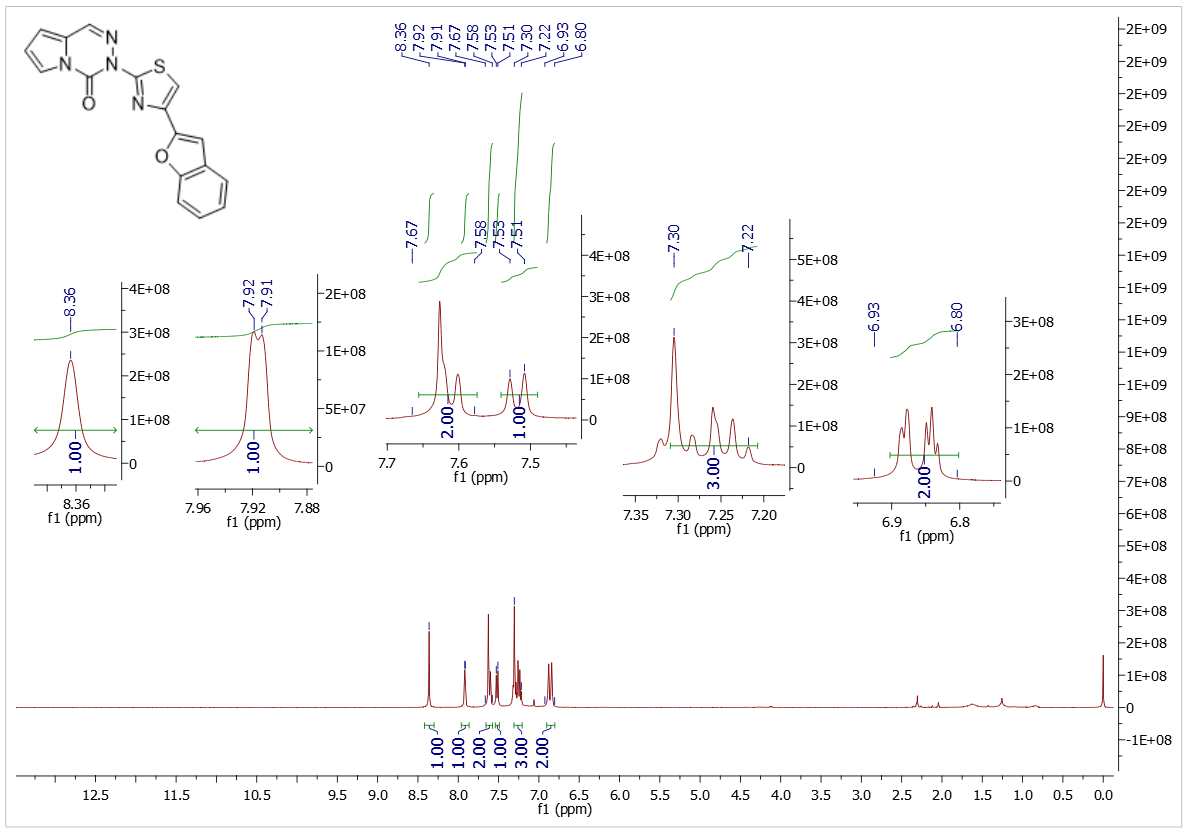


**Figure S35.** ^1^H NMR spectrum of compound **30**


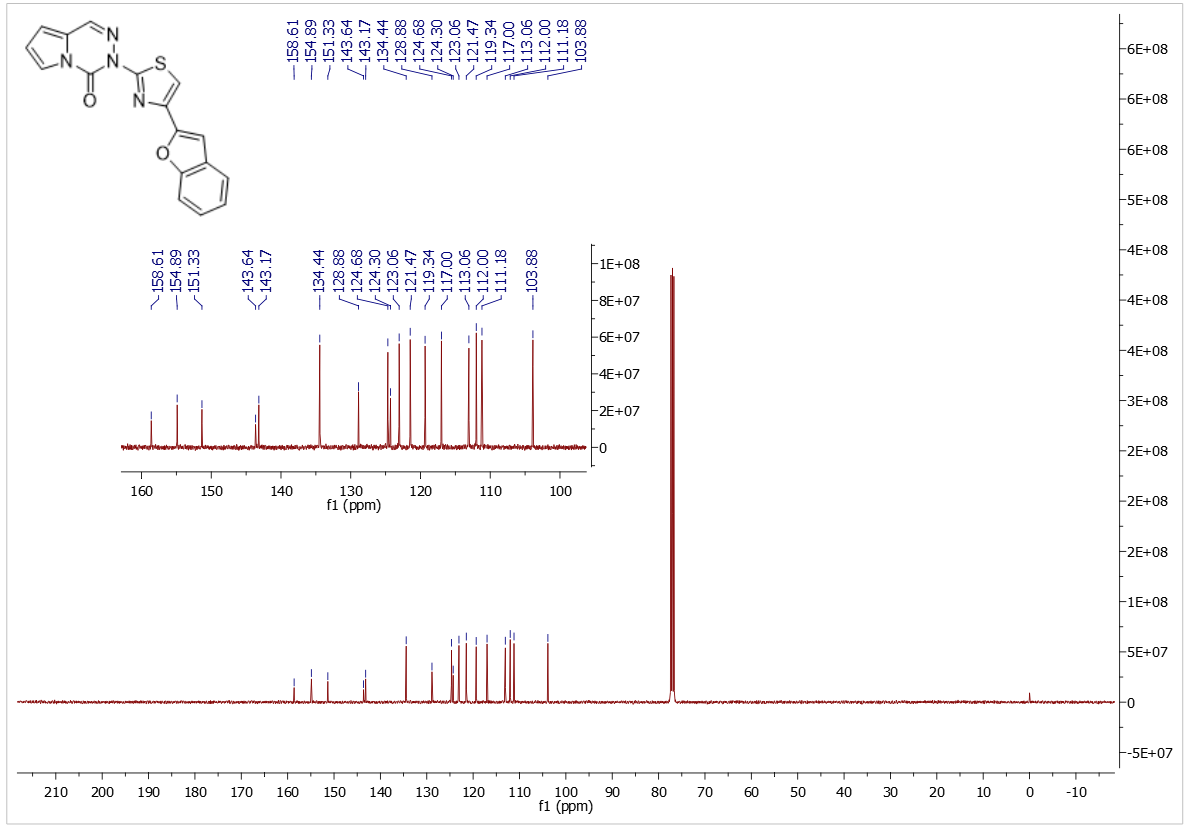


**Figure S36.** ^13^C NMR spectrum of compound **30**


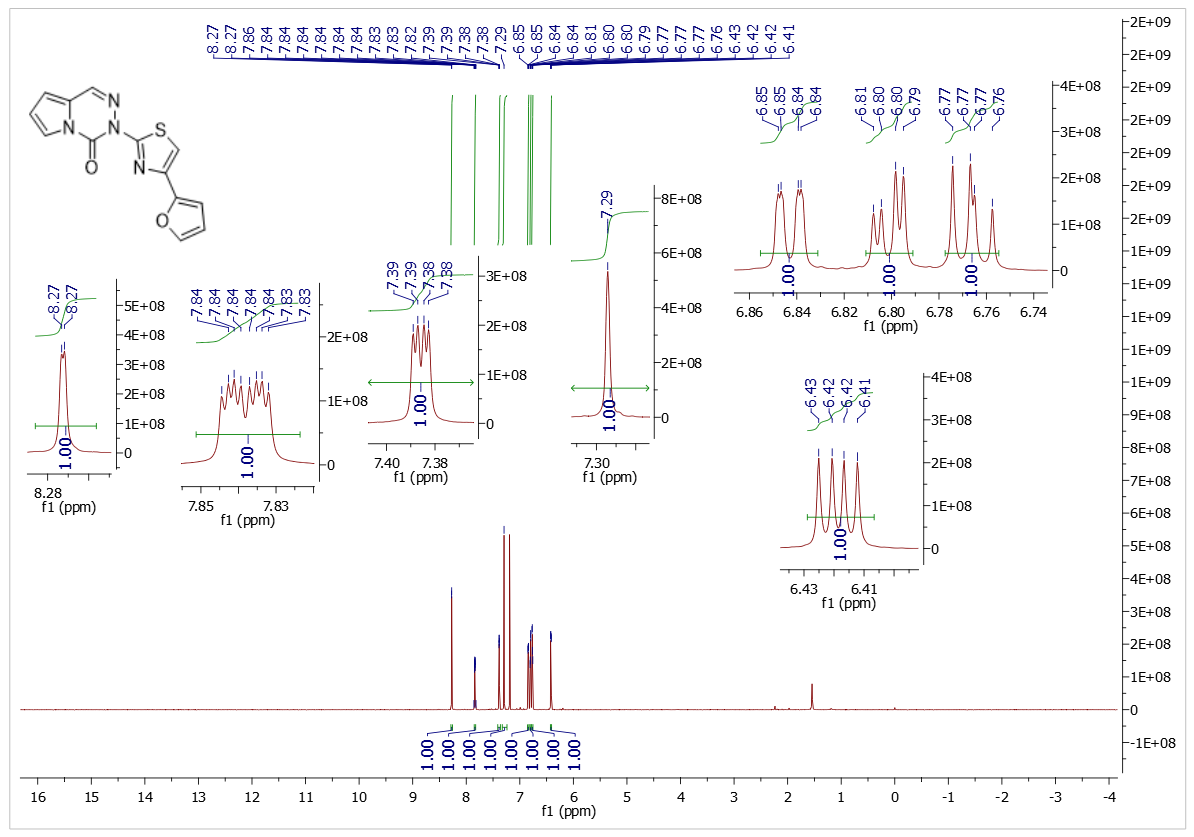


**Figure S37.** ^1^H NMR spectrum of compound **31**


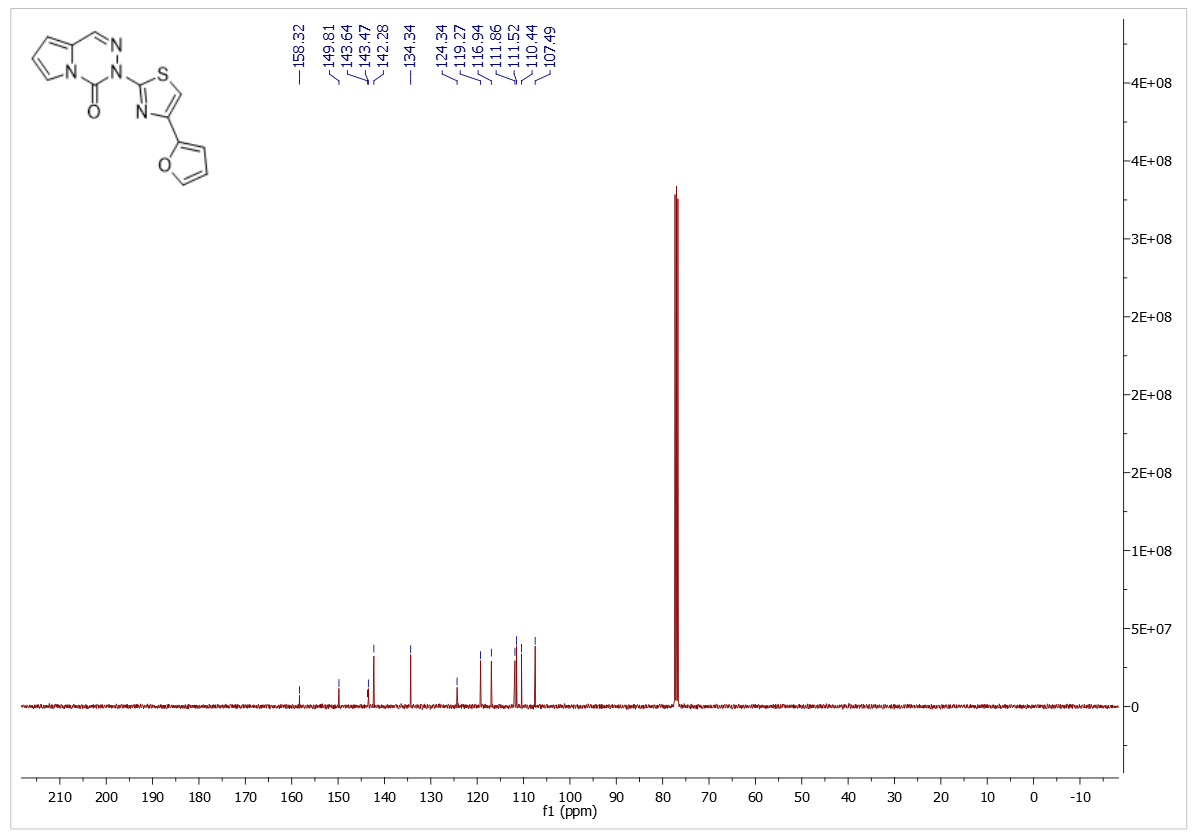


**Figure S38.** ^13^C NMR spectrum of compound **31**


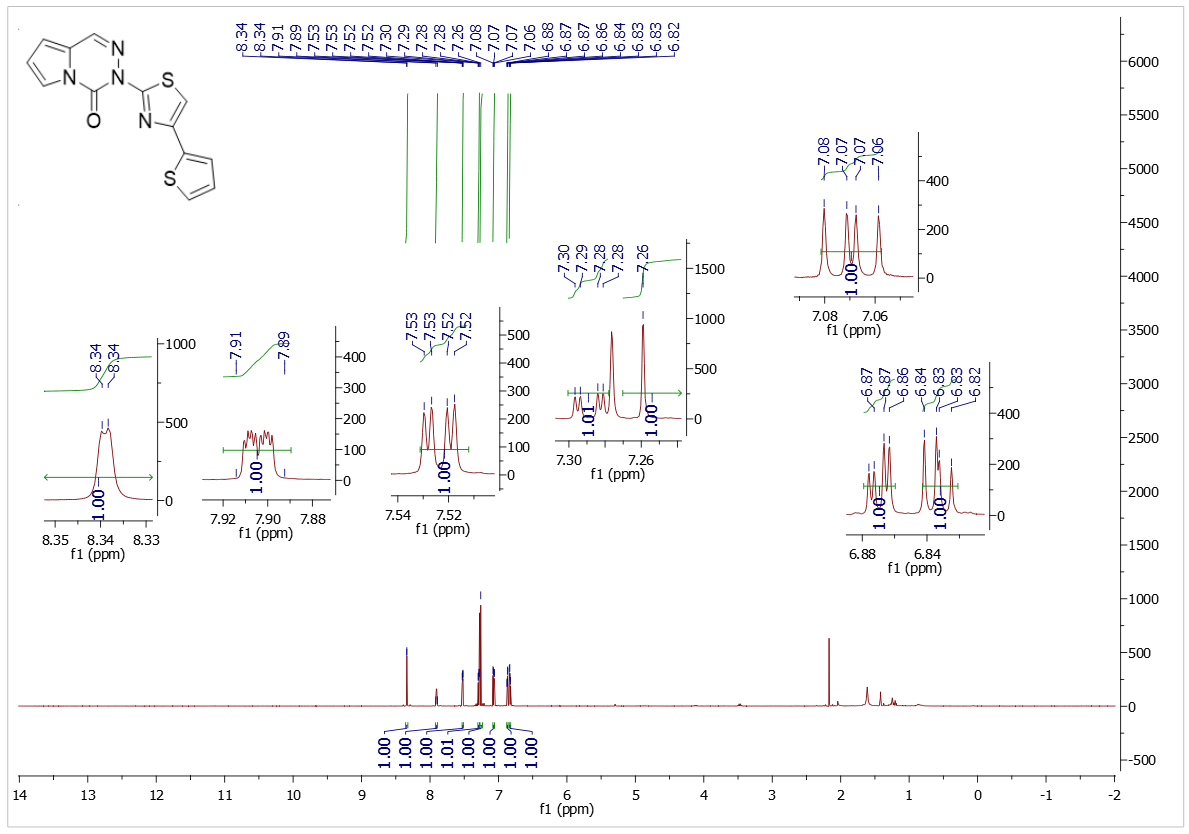


**Figure S39.** ^1^H NMR spectrum of compound **32**


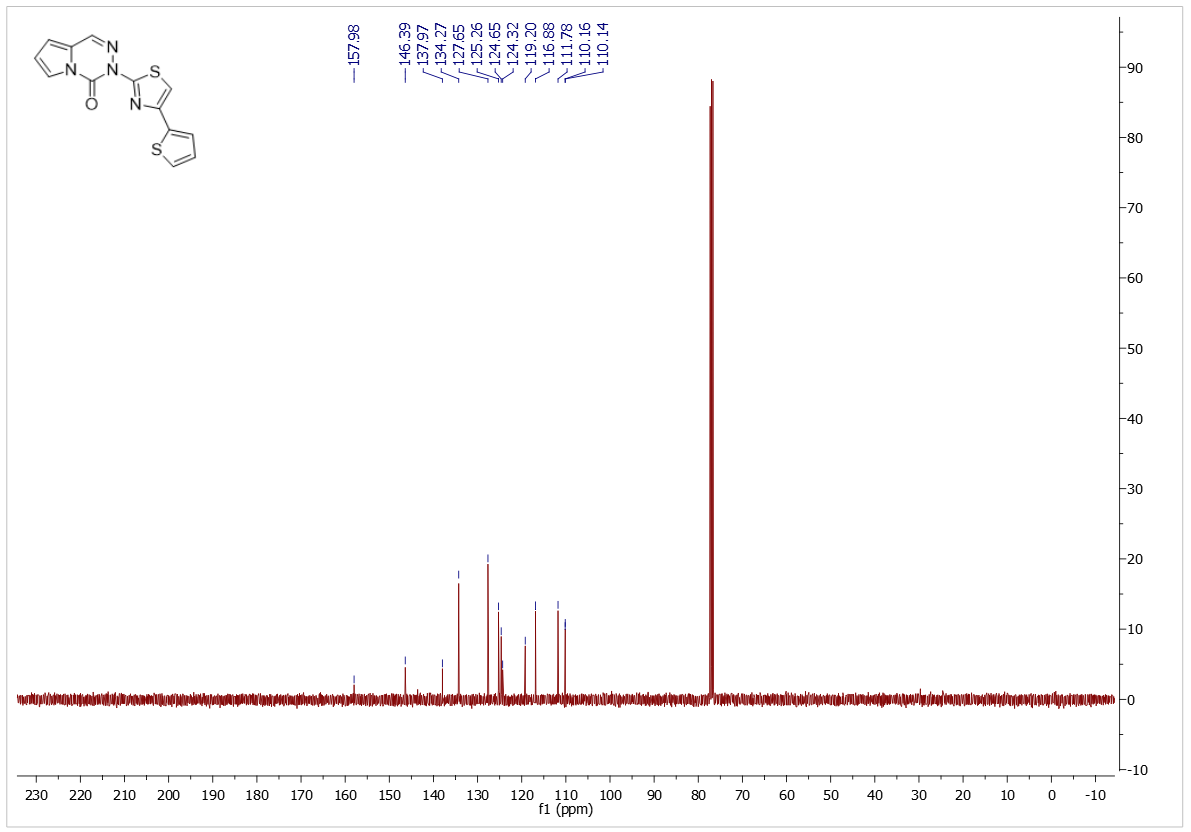


**Figure S40.** ^13^C NMR spectrum of compound **32**

- - - 1. 3D and 2D ligand-protein interactions of PI3K active site with compound **LASW1579**

- - - 1. 3D and 2D ligand-protein interactions of PI3K active site with compound **21**

- - - 1. 3D and 2D ligand-protein interactions of PI3K active site with compound **26**

- - - 1. 3D and 2D ligand-protein interactions of PI3K active site with compound **27**

- - - 1. HRMS spectrum copies of compounds **13-32**

**Figure S41.** HRMS spectrum of compound **13**

**Figure S42.** HRMS spectrum of compound **14**

**Figure S43.** HRMS spectrum of compound **15**

**Figure S44.** HRMS spectrum of compound **16**

**Figure S45.** HRMS spectrum of compound **17**

**Figure S46.** HRMS spectrum of compound **18**

**Figure S47.** HRMS spectrum of compound **19**

**Figure S48.** HRMS spectrum of compound **20**

**Figure S49.** HRMS spectrum of compound **21**

**Figure S50.** HRMS spectrum of compound **22**

**Figure S51.** HRMS spectrum of compound **23**

**Figure S52.** HRMS spectrum of compound **24**

**Figure S53.** HRMS spectrum of compound **25**

**Figure S54.** HRMS spectrum of compound **26**

**Figure S55.** HRMS spectrum of compound **27**

**Figure S56.** HRMS spectrum of compound **28**

**Figure S57.** HRMS spectrum of compound **29**

**Figure S58.** HRMS spectrum of compound **30**

**Figure S59.** HRMS spectrum of compound **31**

**Figure S60.** HRMS spectrum of compound **32**
